# Exploring the HIV-1 Rev Recognition Element (RRE)–Rev Inhibitory Capacity and Antiretroviral Action of Benfluron Analogs

**DOI:** 10.3390/molecules28207031

**Published:** 2023-10-11

**Authors:** Sergi Chumillas, Saurabh Loharch, Manuela Beltrán, Mateusz P. Szewczyk, Silvia Bernal, Maria C. Puertas, Javier Martinez-Picado, José Alcamí, Luis M. Bedoya, Vicente Marchán, José Gallego

**Affiliations:** 1 Departament de Química Inorgànica i Orgànica, Secció de Química Orgànica, IBUB, Universitat de Barcelona, 08028 Barcelona, Spain; sergichv@gmail.com; 2 Centro de Investigación Traslacional San Alberto Magno, Universidad Católica de Valencia San Vicente Mártir, 46001 Valencia, Spain; saurabh.loharch@ucv.es (S.L.); mateusz.szewczyk@ucv.es (M.P.S.); 3 Instituto de Salud Carlos III, 28220 Majadahonda, Spain; mbeltran@isciii.es (M.B.); lmbedoya@ucm.es (L.M.B.); 4 CIBERINFEC, Instituto de Salud Carlos III, 28029 Madrid, Spain; 5 Escuela de Doctorado, Universidad Católica de Valencia San Vicente Mártir, 46001 Valencia, Spain; 6 IrsiCaixa AIDS Research Institute, 08916 Badalona, Spain; 7 Infectious Diseases and Immunity Department, University of Vic—Central University of Catalonia, 08500 Vic, Spain; 8 Germans Trias i Pujol Research Institute, 08916 Badalona, Spain; 9 Catalan Institution for Research and Advanced Studies, 08010 Barcelona, Spain; 10 Facultad de Farmacia, Universidad Complutense de Madrid, 28040 Madrid, Spain

**Keywords:** antiviral drug discovery, benfluron, benzo[c]fluorenone, human immunodeficiency virus type 1, Rev, RNA

## Abstract

Human immunodeficiency virus-type 1 (HIV-1) remains one of the leading contributors to the global burden of disease, and novel antiretroviral agents with alternative mechanisms are needed to cure this infection. Here, we describe an exploratory attempt to optimize the antiretroviral properties of benfluron, a cytostatic agent previously reported to exhibit strong anti-HIV activity likely based on inhibitory actions on virus transcription and Rev-mediated viral RNA export. After obtaining six analogs designed to modify the benzo[c]fluorenone system of the parent molecule, we examined their antiretroviral and toxicity properties together with their capacity to recognize the Rev Recognition Element (RRE) of the virus RNA and inhibit the RRE–Rev interaction. The results indicated that both the benzo[c] and cyclopentanone components of benfluron are required for strong RRE–Rev target engagement and antiretroviral activity and revealed the relative impact of these moieties on RRE affinity, RRE–Rev inhibition, antiviral action and cellular toxicity. These data provide insights into the biological properties of the benzo[c]fluorenone scaffold and contribute to facilitating the design of new anti-HIV agents based on the inhibition of Rev function.

## 1. Introduction

**Figure 1:**
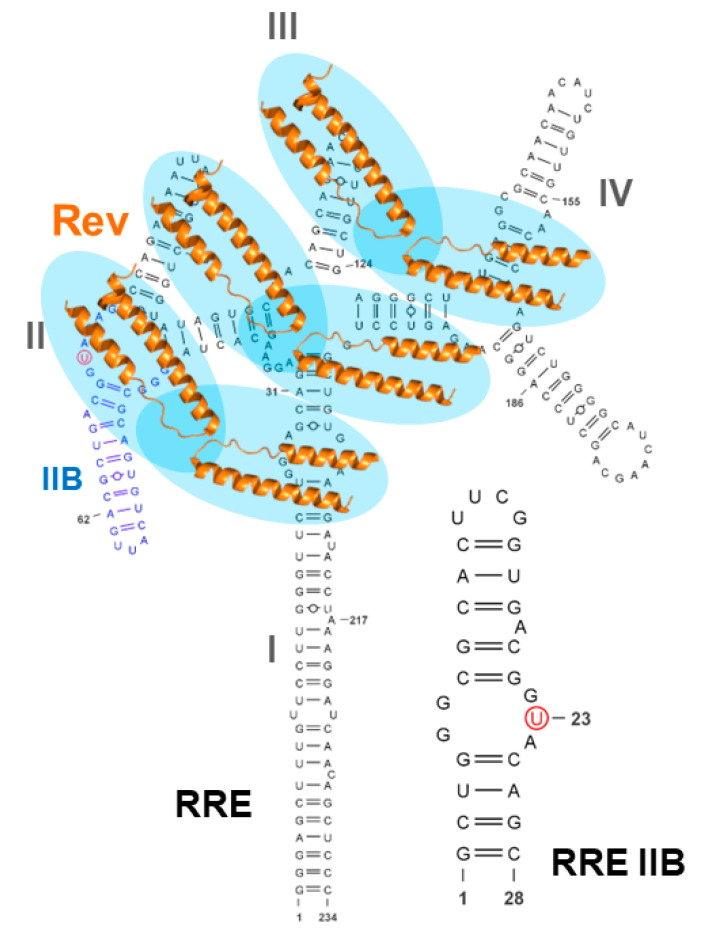
The RRE–Rev complex of HIV-1. Secondary structure model of the full-length RRE–Rev complex, where several Rev monomers (encircled with blue ovals) bind to the RRE RNA structure. The location of RRE subdomain IIB, forming the main Rev-binding site, is indicated with blue lettering. The secondary structure of hairpin RRE IIB, containing the high-affinity Rev-binding site, is shown on the right. The extrahelical loop nucleotide U23 of this hairpin (marked with a red circle) was labeled with fluorescein for fluorescence binding assays.

**Figure 2:**
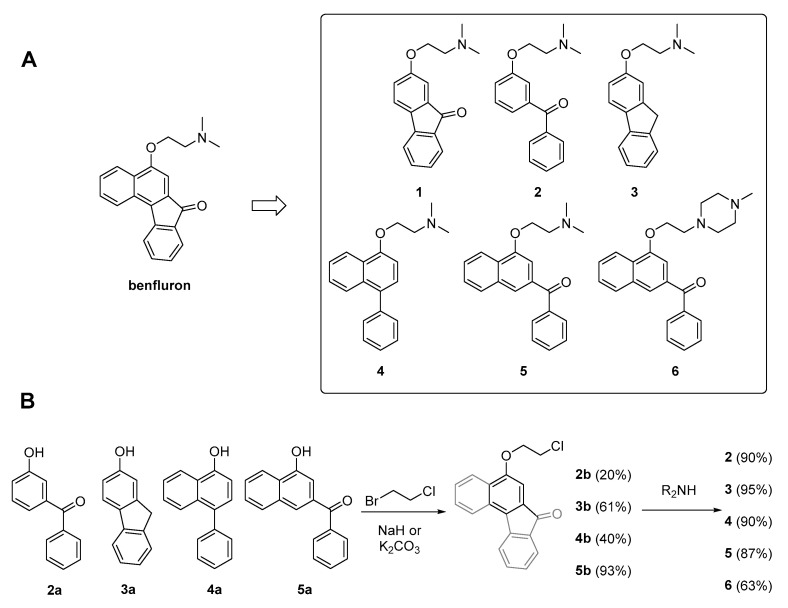
Chemical structure of benfluron and of analogs **1**–**6** investigated in this report (**A**), and general procedure for the synthesis of compounds **2**–**6** from the corresponding phenol or naphthol precursors **2a**–**5a** (**B**).

The current antiretroviral therapy is not curative, making the discovery of new anti-HIV agents with alternative mechanisms of action a necessity [1]. Among the possible targets for direct action not used by presently marketed antiretrovirals, the complex formed between the virus-encoded protein Rev and the RRE of the viral RNA genome remains unexploited despite different efforts carried out to develop inhibitors (e.g., [2,3,4,5,6,7,8,9,10,11]). This ribonucleoprotein complex comprises several Rev protein monomers bound to the RRE RNA structure (Figure 1) and enables the nuclear export of unspliced or singly spliced HIV-1 transcripts, an essential step in the virus replication cycle [12]. By screening a library of drug-like compounds with an assay based on monitoring the interaction between Rev and its high-affinity binding site in RRE subdomain IIB, we recently identified benfluron as an RRE–Rev inhibitor [13] (Figure 2A). This benzo[c]fluorenone compound was shown to be capable of recognizing RRE subdomain IIB and inhibiting the formation of the full-length RRE–Rev ribonucleoprotein complex. On the other hand, benfluron blocked HIV-1 replication in MT-2 cells with a submicromolar EC_50_ of 0.830 µM and a cytotoxic concentration, CC_50_, of 28.1 µM, resulting in a selectivity index (SI; defined as CC_50_/EC_50_) of 33.9 in this cell type [13]. Further RT-PCR and transfection experiments indicated that the mechanism of antiviral action of this compound was based on the inhibition of Rev function and viral transcription [13]. Altogether, these findings made benfluron an attractive candidate for a chemical optimization process aimed at identifying new Rev-based antiretroviral agents. Here, we report an exploratory optimization of this molecule and describe the impact of the benzo[c] and cyclopentanone components of benfluron on RRE–Rev target engagement, antiviral action and cellular toxicity.

## 2. Results

### 2.1. Optimization Strategy

The fact that benfluron had been previously studied as a cytostatic agent [14] suggested that the therapeutic index of the compound could be a key consideration for optimization. To test this hypothesis, we first evaluated the selectivity of the molecule in peripheral blood mononuclear cells (PMBCs), the natural host of the virus, obtaining antiviral EC_50_ and cytotoxic CC_50_ values of 5.5 and of 7.1 µM, respectively (Figure S1). This implied an SI of 1.3, substantially lower than that obtained in MT-2 cells [13]. Based on this result, the chemical optimization was focused on lowering toxicity while maintaining or improving RRE–Rev-inhibition capacity and antiretroviral activity. Since the fluorenone moiety of benfluron is present in several DNA-binding molecules [15] and this could be associated with intercalation between base-pairs and toxicity, we aimed to investigate the effect of reducing either the planarity of the aromatic system or the number of fused rings within the benzo[c]fluorenone system of benfluron. To achieve this goal, we designed six benfluron analogs (compounds **1**–**6** in Figure 2A) and measured the effect on RRE affinity, RRE–Rev-inhibition capacity, antiretroviral activity and cellular toxicity.

### 2.2. Benfluron Analogs

Benfluron analogs **1**–**5** retained the *N*,*N*-dimethylaminoethoxy chain of the parent molecule, which was similarly oriented relative to the aromatic system of the molecules, whereas analog **6** incorporated an *N*-methylpiperazine heterocycle that provided an additional group suitable for electrostatic interactions (Figure 2A).

**Scheme 1:**
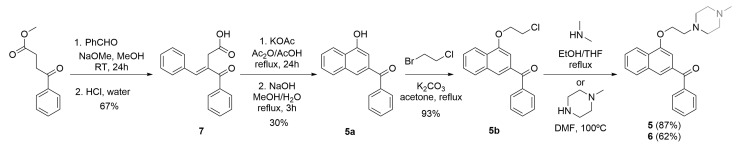
Synthesis of benfluron analogs **5** and **6**.

The fluorenone analog lacking the benzene[c] ring (**1**) was obtained commercially, whereas compounds **2**–**6** were synthesized following the general procedure shown in Figure 2B. This approach involved alkylation of suitable phenol (**2a**–**3a**) or naphthol (**4a**–**5a**) derivatives with 1-bromo-2-chloroethane under basic conditions, followed by a nucleophilic bimolecular substitution of the chlorinated intermediates (**2b**–**5b**) with dimethylamine or *N*-methyl-piperazine, which provided benfluron analogs **2**–**6** with good yields. While phenols **2a** and **3a** were commercially available, 1-naphthol derivatives **4a** and **5a** had to be synthetically obtained. On the one hand, the synthesis of **4a** involved a Suzuki cross-coupling reaction between 4-bromo-naphthol and phenylboronic acid, which afforded the desired product with a 60% reaction yield after purification via silica column chromatography. The synthesis of **5a** (Scheme 1), on the other hand, first involved an aldol condensation between methyl 4-oxo-4-phenylbutanoate and benzaldehyde to provide **7**, which was subjected to an acetic anhydride-promoted intramolecular Friedel–Crafts acylation to obtain **5a** with a 30% yield. All compounds were fully characterized using ^1^H and ^13^C NMR, as well as via high-resolution MS, and their purity was assessed through reversed-phase HPLC analysis (Figures S2–S30).

### 2.3. RRE Affinity and RRE–Rev Inhibition

**Figure 3:**
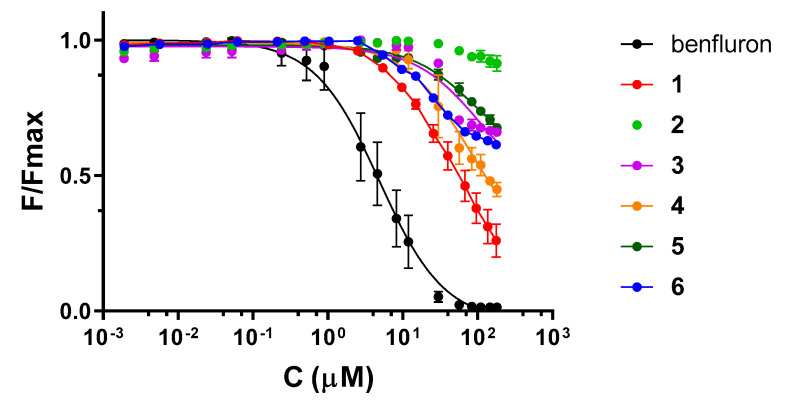
RRE subdomain IIB recognition of benfluron and benfluron analogs **1**–**6**. The graph shows hairpin RRE IIB association curves obtained with fluorescence binding experiments. The error bars represent the standard deviation of at least three independent experiments.

**Figure 4:**
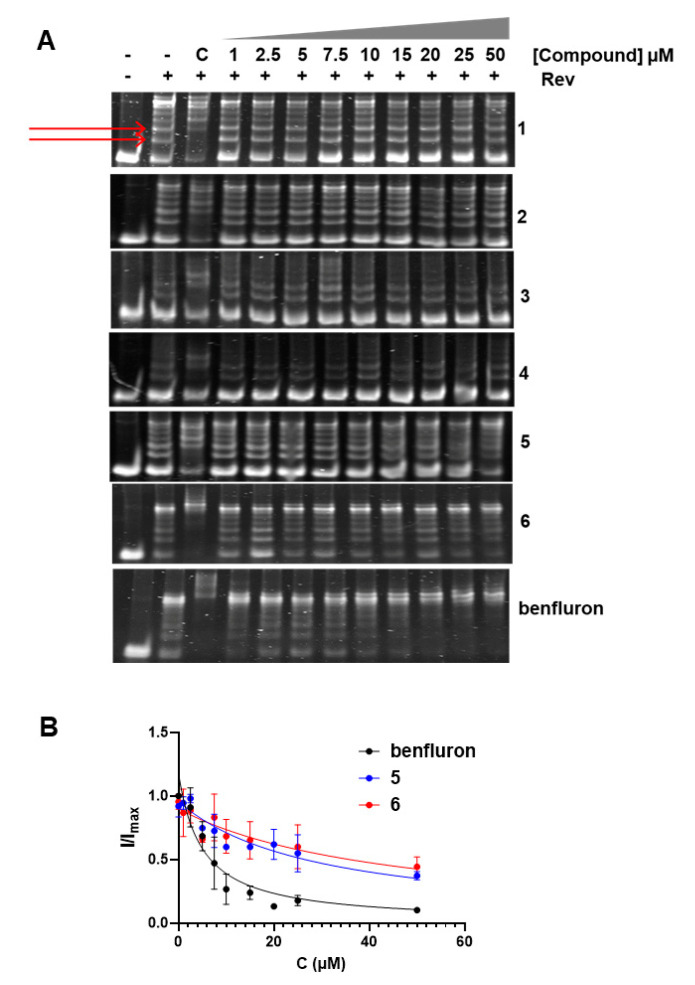
RRE–Rev-inhibition capacity of benfluron and benfluron analogs **1**–**6**. (**A**) Inhibition of full-length RRE–Rev complex formation analyzed via EMSA. The gel lanes contained 70 nM RRE, 0.75 μM Rev (marked with +), and the specified concentrations of benfluron and analogs **1**–**6**. An inhibition control lane containing 50 µM benfluron was included in the EMSA of each analog (labelled with C). For benfluron, the inhibition control contained 50 µM of the RRE–Rev inhibitor bisphenyl-indol [16]. The arrows in the first EMSA indicate the low-order RRE–Rev bands used for quantification. A representative experiment of three independent replicates is shown for each compound. (**B**) Plots of low-order RRE–Rev EMSA band intensity as a function of compound concentration for benfluron and active analogs **5** and **6**; the error bars represent the standard deviation of three independent experiments.

To assess putative target engagement, we measured with fluorescence experiments compound binding to RRE subdomain IIB, the high-affinity binding site of Rev within the RRE, as well as inhibition of full-length RRE–Rev complex formation with electrophoretic mobility shift assays (EMSAs). The *N*,*N*-dimethylaminoethoxy-fluorenone analog **1** lacking the benzene[c] ring of benfluron (Figure 2A) exhibited lesser RRE subdomain IIB affinity relative to the parent molecule (K_d_ = 45.6 vs. 4.83 µM) and did not exhibit full-length RRE–Rev-inhibition capacity at concentrations below 50 µM (Table 1 and Figure 3 and Figure 4). A yet stronger deleterious impact was observed for two other analogs lacking either the cyclopentanone ring (**2**) or the carbonyl group (**3**) in addition to the benzene[c] ring (Figure 2A). These two compounds likewise lost RRE–Rev inhibition at concentrations below 50 µM and exhibited even less affinity for RRE subdomain IIB (Table 1 and Figure 3 and Figure 4).

Compared to **2**, compound **5**, maintaining the naphthalene moiety of benfluron but similarly lacking the cyclopentanone ring (Figure 2A), had somewhat better RRE subdomain IIB affinity and retained full-length RRE–Rev inhibition at approximately 29 µM. However, RRE IIB affinity and RRE–Rev-inhibition capacity were still significantly poorer compared with those of the parent molecule (Table 1 and Figure 3 and Figure 4). The naphthalene analog **6**, similarly lacking the fluorenone moiety but incorporating a piperazine heterocycle in the ethoxy chain (Figure 2A), exhibited higher RRE IIB affinity relative to **5**, but still had weak RRE–Rev-inhibition capacity at approximately 45 µM. Related naphthalene analog **4**, where the cyclopentanone ring was removed and planarity was partially lost, also had weak RRE subdomain IIB affinity and lacked RRE–Rev inhibition at concentrations below 50 µM (Table 1 and Figure 3 and Figure 4). Overall, these results indicated that both the benzene[c] and cyclopentanone rings of benfluron are important structural motifs for RRE subdomain IIB binding and RRE–Rev inhibition. On a more detailed level, a comparison of the K_d_ values of benfluron and analogs **1**, **4** and **5** and the K_d_ values of compounds **5** and **2** revealed that the cyclopentanone moiety was particularly important for subdomain IIB RNA association. Conversely, RRE–Rev inhibition was only observed for analogs **5** and **6** retaining the benzene[c] ring (Table 1 and Figure 2A, Figure 3 and Figure 4).

### 2.4. Antiretroviral Activity and Toxicity

**Figure 5:**
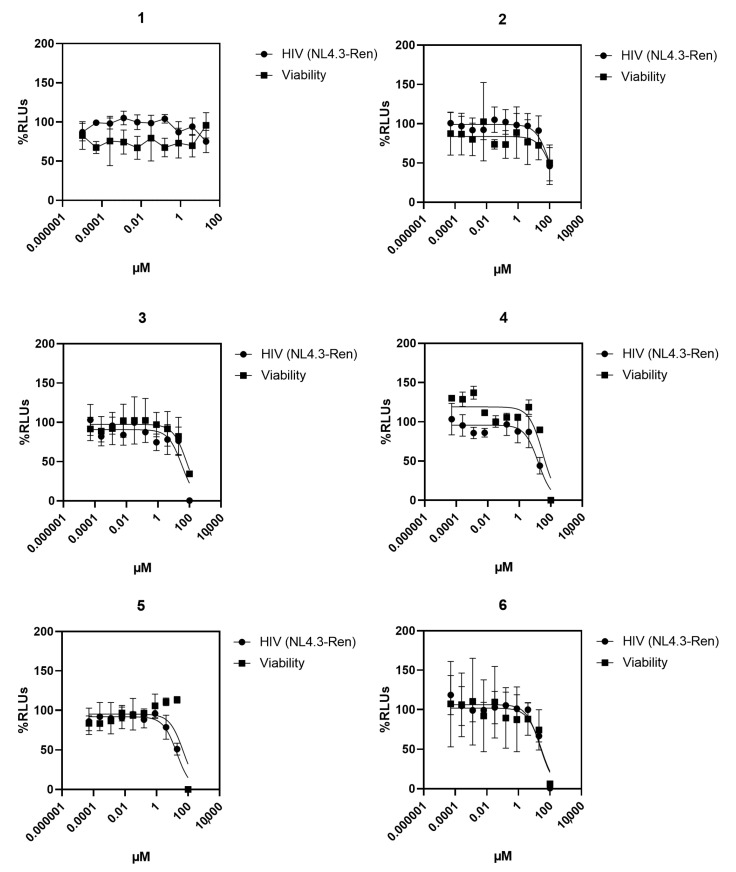
Antiretroviral activity and cellular toxicity of benfluron analogs **1**–**6** in MT-2 cell culture. Antiviral activity is expressed as a percentage of relative luminescence units (RLUs), where 100% is the level of viral replication obtained in the presence of the vehicle used to dissolve the compounds.

The removal of the benzene[c] ring in analogs **1**–**3** eliminated cellular toxicity at most assay concentrations but also had a strong deleterious impact on anti-HIV activity (Table 2 and Figure 5). Restoring the benzene[c] ring while eliminating the cyclopentanone ring of the fluorenone system, as in compounds **4**–**6**, partially recovered the antiretroviral activity. The EC_50_ values were significant (between 17 and 24 µM), but well above that of benfluron, 0.83 µM. However, this also brought about some cellular toxicity, indicated by CC_50_ values between 20 and 49.7 µM. Compound **5**, with an anti-HIV EC_50_ of 19.4 µM and a CC_50_ of 49.7 µM (SI = 2.6), was the best compound of the optimization series (Table 2 and Figure 5). Overall, a comparison of the cellular data of benfluron and analogs **1**–**6** indicated that both the benzene[c] and cyclopentanone rings of the parent molecule were required for strong antiretroviral activity. The naphthalene system was a major contributor to anti-HIV action but also an important determinant of cellular toxicity, whereas removal of the fluorenone cyclopentanone ring had a lesser impact on both the antiviral activity and toxicity.

## 3. Discussion

We recently reported that the cytostatic agent benfluron had significant antiretroviral activity, likely based on inhibitory actions on virus transcription and RRE–Rev-mediated RNA nuclear export [13]. Here, we describe an exploratory attempt to optimize the antiretroviral and toxicity properties of this agent, focused on reducing the size and planarity of the benzo[c]fluorenone aromatic system in order to decrease putative intercalation between base-pairs. After monitoring the RRE–Rev-target-recognition capacity and the antiretroviral activity and cellular toxicity of six chemical analogs lacking either the benzene[c] and/or cyclopentanone components of benfluron, we conclude that both moieties seem to be required for strong RRE–Rev inhibition and antiviral activity. The cyclopentanone ring significantly increased RRE affinity in vitro and anti-HIV activity in cells. The benzene[c] ring, on the other hand, was crucial for antiretroviral activity and likely promoted RRE–Rev inhibition, but also acted as a driver of cellular toxicity. The antiretroviral activities measured in cells roughly followed the trend observed for RRE binding and RRE–Rev inhibition, since both the benzene[c] and cyclopentanone moieties were required for a strong effect on the RNA and protein species. However, we cannot disregard a possible action on other targets. In this regard, we reported a strong action on viral transcription, in addition to Rev function, for benfluron [13].

Although compounds **4**–**6**, keeping the benzene[c] component of benfluron, exhibited significant antiretroviral activity and **5** tended to be less toxic than the parent molecule, none of the analogs improved the activity of benfluron. However, the experiments described in this report revealed the relative impact of the benzo[c] and cyclopentanone components of the benzo[c]fluorenone system on RNA recognition, RRE–Rev inhibition, antiviral action and toxicity. These data contribute to clarifying the mechanism of antiviral action of the benfluron scaffold and will be useful for future optimization attempts.

## 4. Materials and Methods

### 4.1. Materials and Methods for Compound Preparation

Unless otherwise stated, common chemicals and solvents (HPLC-grade or reagent-grade quality) were purchased from commercial sources and used without further purification. A hot plate magnetic stirrer, together with an aluminum reaction block of the appropriate size, was used as the heating source in all reactions requiring heat. Aluminum plates coated with a 0.2 mm-thick layer of silica gel 60 F_254_ were used for thin-layer chromatography analyses (TLC), whereas column chromatography purification was carried out using silica gel 60 (230–400 mesh). Reversed-phase high-performance liquid chromatography (HPLC) analyses were carried out on a Jupiter Proteo C_12_ column (150 × 4.6 mm, 90 Å 4 µm; flow rate: 1 mL/min) using linear gradients of 0.1% formic acid in H_2_O (A) and 0.1% formic acid in ACN (B). Gas chromatography analyses were carried out on a HP6890 Series Instrument equipped with a 5973 Mass Selective detector with an EI as an ionization source and an HP-5MS UI column of 30 m long, and a 0.250 mm diameter was used with helium as the mobile phase. NMR spectra were recorded at 25 °C in 400 or 500 MHz spectrometers using the deuterated solvent as an internal deuterium lock. The residual protic signal of chloroform or DMSO was used as a reference in the ^1^H and ^13^C NMR spectra recorded in CDCl_3_ or DMSO-*d_6_*, respectively. Chemical shifts are reported in parts per million (ppm) on the δ scale, coupling constants in Hz and multiplicity as follows: s (singlet), d (doublet), t (triplet), q (quartet), qt (quintuplet), m (multiplet), dd (doublet of doublets), dq (doublet of quartets), br (broad signal), etc. Electrospray ionization mass spectra (ESI-MS) were recorded on an instrument equipped with a single quadrupole detector coupled to an HPLC and high-resolution (HR) ESI-MS on an LC/MS-TOF instrument. Compound **1** was purchased from Enamine. Stock solutions of all compounds were prepared in DMSO at a 20 mM concentration.

### 4.2. Synthesis of Benfluron Analogs 2–6

*Compound **2b**.* 3-Hydroxy-benzophenone (200 mg, 1.01 mmol) and potassium tert-butoxide (120 mg, 1.07 mmol) were dissolved in anhydrous THF (20 mL) under an Ar atmosphere. The resulting yellowness solution was stirred at room temperature for 5 min. Then, 1-bromo-2-chloroethane (167 µL, 2.02 mmol) was added, and the resulting solution was refluxed overnight under an Ar atmosphere. The solvent was evaporated under reduced pressure, and the product was isolated via column chromatography (silica gel, 0–10% ethyl acetate in hexanes). Then, 54 mg of colorless oil was obtained (yield: 21%). TLC: Rf (30% ethyl acetate in hexanes) 0.8. ^1^H NMR (400 MHz, CDCl_3_) δ (ppm): 7.79 (m, 2H), 7.59 (m, 1H), 7.48 (m, 2H), 7.38 (m, 3H), 7.17 (m, 1H), 4.29 (t, *J* = 5.8 Hz, 2H), 3.83 (t, *J* = 5.8 Hz, 2H). ^13^C NMR (101 MHz, CDCl_3_) δ (ppm): 196.4, 158.3, 139.1, 137.6, 132.6, 130.1, 129.5, 128.4, 123.6, 119.6, 115.2, 77.5, 77.2, 76.8, 68.3, 42.0. HR-ESI MS (ESI-TOF, positive mode): *m*/*z* 261.0677 calc. for [C_15_H_13_ClO_2_ + H]^+^: 261.0673.

Compound **2**. To a solution of **2b** (100 mg, 0.384 mmol) in DMF (2 mL), dimethylamine (1 mL, 2 M in THF) was added, and the resulting mixture was refluxed overnight. Then, the crude was diluted with ethyl acetate (50 mL) and washed with water (3 × 50 mL). The organic layer was dried over anhydrous MgSO_4_, filtered and evaporated to dryness under reduced pressure. The product was isolated via column chromatography (silica gel, 0–10% MeOH in DCM) to provide 92 mg of an orange oil (yield: 90%). TLC: Rf (10% MeOH in DCM) 0.4. ^1^H NMR (500 MHz, CDCl_3_) δ (ppm): 7.79 (m, 2H), 7.58 (m, 1H), 7.48 (m, 2H), 7.36 (s, 3H), 7.16 (m, 1H), 4.13 (t, *J* = 5.6 Hz, 2H), 2.77 (t, *J* = 5.6 Hz, 2H), 2.36 (s, 6H). ^13^C NMR (126 MHz, CDCl_3_) δ (ppm): 196.7, 159.0, 139.0, 137.8, 132.5, 130.2, 129.4, 128.4, 123.1, 119.6, 115.2, 66.3, 58.4, 46.0. HR-ESI MS (ESI-TOF, positive mode): *m/z* 270.1489 calc. for [C_17_H_19_NO_2_ + H]^+^: 270.1485.

Compound **3b**. A solution of 3-hydroxyfluorene (15 mg, 0.082 mmol) and NaH (60% dispersion in mineral oil, 17 mg, 0.412 mmol) in anhydrous THF (5 mL) was stirred for 1 h at room temperature under an Ar atmosphere. Then, 1-bromo-2-chloroethane (68 µL, 0.824 mmol) was added, and the mixture was refluxed overnight under Ar. After evaporation under reduced pressure, the product was isolated via column chromatography (silica gel, 0–30% ethyl acetate in hexanes). Then, 12 mg of a colorless oil was obtained (yield: 61%). TLC: Rf (30% ethyl acetate in hexanes) 0.8. ^1^H NMR (500 MHz, CDCl_3_) δ (ppm): 7.69 (t, *J* = 7.8 Hz, 2H), 7.51 (d, *J* = 7.4 Hz, 1H), 7.35 (t, *J* = 7.4 Hz, 1H), 7.25 (t, *J* = 7.7 Hz, 1H), 7.12 (s, 1H), 6.95 (dd, *J* = 8.3, 2.2 Hz, 1H), 4.29 (t, *J* = 5.9 Hz, 2H), 3.87 (s, 2H), 3.85 (t, *J* = 5.9 Hz, 2H). ^13^C NMR (126 MHz, CDCl_3_) δ (ppm): 157.9, 145.3, 142.9, 141.6, 135.6, 126.9, 125.9, 125.0, 120.7, 119.3, 113.9, 111.8, 68.5, 42.1, 37.1. HR-ESI MS (ESI-TOF, positive mode): *m*/*z* 245.0728 calc. for [C_15_H_13_ClO + H]^+^: 245.0722.

Compound **3**. To a solution of **3b** (20 mg, 0.082 mmol) in absolute ethanol (1 mL), dimethylamine (1 mL, 2 M in THF) was added, and the resulting mixture was refluxed for 3 days. After evaporation under reduced pressure, purification was carried out via column chromatography (silica gel, 0–10% MeOH in DCM) to obtain 25 mg of an orange oil (yield: 95%). TLC: Rf (10% MeOH/DCM) 0.5. ^1^H NMR (400 MHz, DMSO-*d_6_*) δ (ppm): 7.81 (t, *J* = 7.8 Hz, 2H), 7.54 (d, *J* = 7.4 Hz, 1H), 7.33 (t, 1H), 7.25 (m, 2H), 7.02 (dd, *J* = 8.4, 2.2 Hz, 1H), 4.30 (t, *J* = 5.2 Hz, 2H), 3.89 (s, 2H), 3.33 (t, J = 5.2 Hz, 2H), 2.73 (s, 6H). ^13^C NMR (101 MHz, DMSO-*d_6_*) δ (ppm): 157.4, 144.9, 142.5, 140.9, 134.6, 126.7, 125.8, 125.0, 120.8, 119.3, 113.9, 111.4, 63.3, 56.1, 43.6, 36.5. HR-ESI MS (ESI-TOF, positive mode): *m*/*z* 254.1539 calc. for [C_17_H_19_NO + H]^+^: 254.1539.

Compound **4a**. 4-Bromo-naphthol (200 mg, 0.897 mmol), phenyl boronic acid (164 mg, 1.345 mmol), sodium hydroxide (143 mg, 3.586 mmol) and [Pd(OAc)_2_]_3_ (20 mg, 0.090 mmol) were dissolved in 4 mL of MilliQ water. The mixture was bubbled up with Ar for 5 min and stirred for two days at room temperature. Then, the crude was extracted with ethyl acetate (3 × 50 mL), and the combined organic phases were dried over anhydrous Na_2_SO_4_, filtered and evaporated to dryness under reduced pressure. The product was isolated via column chromatography (silica gel, 0–10% ethyl acetate in hexanes), obtaining 118 mg of a white solid (yield: 60%). TLC: Rf (20% ethyl acetate in hexanes) 0.4. ^1^H NMR (400 MHz, CDCl_3_) δ (ppm): 8.26 (d, *J* = 9.6 Hz, 1H), 7.87 (d, *J* = 8.9 Hz, 1H), 7.47 (m, 7H), 7.25 (d, *J* = 7.7 Hz, 1H), 6.86 (d, *J* = 7.7 Hz, 1H). ^13^C NMR (101 MHz, CDCl_3_) δ (ppm): 151.0, 140.9, 133.4, 132.8, 130.4, 128.4, 127.1, 127.0, 126.7, 126.1, 125.3, 124.6, 122.0, 108.3. HR-ESI MS (ESI-TOF, negative mode): *m*/*z* 219.0815 calc. for [C_16_H_12_O-H]^−^: 219.0817.

Compound **4b**. To a suspension of **4a** (50 mg, 0.227 mmol) and potassium carbonate (94 mg, 0.341 mmol) in acetone (1 mL), 1-bromo-2-chloroethane (94 µL, 1.136 mmol) was added under an Ar atmosphere, and the resulting mixture was refluxed for 3 days. Then, the crude solution was diluted with water and extracted with ethyl acetate (3 × 50 mL). The combined organic phases were dried over anhydrous MgSO_4_, filtered and evaporated to dryness under reduced pressure. The product was isolated via column chromatography (silica-gel, 0–20% ethyl acetate in hexanes) to obtain 26 mg of gray oil (Yield 40%). TLC: Rf (20% ethyl acetate in hexanes) 0.5. ^1^H NMR (400 MHz, CDCl_3_) δ (ppm): 8.40 (dd, *J* = 8.3, 2.1 Hz, 1H), 7.87 (dd, *J* = 8.0, 1.7 Hz, 1H), 7.48 (m, 7H), 7.33 (d, *J* = 7.8 Hz, 1H), 6.87 (d, *J* = 7.9 Hz, 1H), 4.47 (t, *J* = 5.8 Hz, 2H), 4.00 (t, *J* = 5.8 Hz, 2H). ^13^C NMR (101 MHz, CDCl_3_) δ (ppm): 153.6, 140.8, 133.7, 132.7, 130.4, 128.4, 127.1, 126.8, 126.8, 126.0, 125.5, 122.4, 104.8, 68.5, 42.2. HR-ESI MS (ESI-TOF, positive mode): *m*/*z* 283.0884 calc. for [C_18_H_15_ClO + H]^+^: 283.0886.

Compound **4**. To a solution of **4b** (10 mg, 0.035 mmol) and potassium iodide (6 mg, 0.035 mmol) in absolute ethanol (1 mL), dimethylamine (1 mL, 2 M in THF) was added, and the resulting mixture was refluxed for 3 days. Then, the solvent was evaporated under reduced pressure, and the crude mixture was partitioned between water (10 mL) and ethyl acetate (10 mL), and the aqueous phase was extracted with ethyl acetate (3 × 10 mL). The combined organic phases were dried over anhydrous MgSO_4_, filtered and evaporated to dryness under reduced pressure. The product was isolated via column chromatography (silica gel, 0–3% MeOH in DCM) to obtain 9 mg of an amorphous orange solid (yield: 90%). TLC: Rf (10% MeOH in DCM) 0.6. ^1^H NMR (400 MHz, DMSO-*d_6_*) δ (ppm): 8.37 (d, *J* = 7.6 Hz, 1H), 7.78 (d, *J* = 8.9 Hz, 1H), 7.54 (m, 7H), 7.37 (d, *J* = 7.9 Hz, 1H), 7.10 (d, *J* = 8.0 Hz, 1H), 4.47 (t, *J* = 5.0 Hz, 2H), 3.41 (t, *J* = 5.0 Hz, 2H), 2.74 (s, 6H). ^13^C NMR (101 MHz, CDCl_3_) δ (ppm): 154.2, 141.9, 133.0, 132.7, 130.4, 128.4, 127.0, 127.0, 126.7, 125.9, 125.9, 125.3, 122.4, 104.5, 66.8, 58.4, 46.1. HR-ESI MS (ESI-TOF, positive mode): *m*/*z* 292.1696 calc. for [C_20_H_20_NO + H]^+^: 292.1695.

Compound **7**. To a solution of sodium (2.4 g, 104.05 mmol) in absolute methanol (50 mL), benzaldehyde (1.06 mL, 10.405 mmol) was added, and the mixture was stirred for 10 min at 0 °C under an Ar atmosphere. Then, methyl 4-oxo-4-phenylbutanoate (1.75 mL, 10.41 mmol) was added dropwise, and the mixture was stirred at room temperature overnight. The reaction was quenched via acidification with concentrated HCl until a pH of 1.0. The crude was partitioned between water (200 mL) and DCM (200 mL), and the aqueous phase was extracted with DCM (3 × 200 mL). The combined organic phases were dried over anhydrous MgSO_4_, filtered and evaporated to dryness, obtaining 1.9 g of a white solid (yield: 68%). The compound was used in the next step without further purification. TLC: Rf (10% MeOH in DCM) 0.4. ^1^H NMR (500 MHz, CDCl_3_) δ (ppm): 7.82 (m, 2H), 7.58 (m, 1H), 7.42 (m, 8H), 3.79 (s, 2H). ^13^C NMR (101 MHz, CDCl_3_) δ (ppm): 198.9, 176.3, 145.5, 137.8, 134.6, 133.6, 132.4, 130.0, 129.5, 129.3, 129.0, 128.5, 34.1. HR-ESI MS (ESI-TOF, negative mode): *m*/*z* 265.0870 calc. for [C_17_H_14_O_3_-H]^−^: 265.0883.

Compound **5a**. Compound **7** (100 mg, 0.375 mmol) and anhydrous sodium acetate (68 mg, 0.824 mmol) were dissolved in a 1:1 (*v*/*v*) mixture of acetic anhydride and acetic acid (1 mL each), and the resulting mixture was refluxed overnight. Then, the crude was neutralized with aqueous 2.5 M NaOH solution, diluted with water (50 mL) and extracted with DCM (4 × 50 mL). The combined organic phases were evaporated to dryness under reduced pressure, and the crude was dissolved in MeOH (2 mL) and aqueous 2.5 M NaOH solution (2 mL), and the resulting mixture was refluxed overnight. Then, the reaction was acidified up to pH 1 with concentrated HCl, diluted with water (50 mL) and extracted with DCM (3 × 50 mL). The combined organic phases were dried over anhydrous MgSO_4_, filtered and evaporated to dryness. Finally, the product was isolated via column chromatography (silica gel, 0–100% DCM in hexanes) to obtain 30 mg of a white solid yield: 32%). TLC: Rf (5% MeOH in DCM) 0.4. ^1^H NMR (500 MHz, CDCl_3_) δ (ppm): 8.31 (d, J = 8.3 Hz, 1H), 7.88 (m, 3H), 7.80 (m, 1H), 7.56 (m, 6H), 7.03 (s, 1H). ^13^C NMR (126 MHz, CDCl_3_) δ (ppm): 151.0, 140.9, 133.4, 132.8, 130.4, 128.4, 127.1, 127.0, 126.7, 126.1, 125.3, 124.6, 122.0, 108.3. HR-ESI MS (ESI-TOF, negative mode): *m*/*z* 247.0765 calc. for [C_17_H_12_O_2_-H]^−^: 247.0763.

Compound **5b**. To a suspension of **5a** (170 mg, 0.685 mmol) and potassium carbonate (947 mg, 6.855 mmol) in acetone (1 mL) under an Ar atmosphere, 1-bromo-2-chloroethane (285 µL, 3.427 mmol) was added, and the mixture was refluxed for 3 days. Then, the solvent was evaporated to dryness, and the crude was partitioned between water (50 mL) and DCM (50 mL), and the aqueous phase was extracted with DCM (3 × 50 mL). The combined organic phases were dried over anhydrous MgSO_4_, filtered and evaporated to dryness. The product was isolated via column chromatography (silica gel, 0–100% DCM in hexanes) to obtain 203 mg of a colorless oil (yield: 93%). TLC: Rf (100% DCM) 0.8. ^1^H NMR (500 MHz, CDCl_3_) δ (ppm): 8.38 (d, *J* = 8.3 Hz, 1H), 7.85 (m, 4H), 7.56 (m, 4H), 7.34 (s, 1H), 4.52 (t, *J* = 5.6 Hz, 3H), 4.00 (t, *J* = 5.6 Hz, 3H). ^13^C NMR (126 MHz, CDCl_3_) δ (ppm): 196.7, 154.6, 138.1, 134.9, 133.3, 132.4, 130.1, 129.2, 128.5, 128.2, 127.9, 127.6, 126.3, 122.4, 104.1, 68.5, 42.2. HR-ESI MS (ESI-TOF, positive mode): *m*/*z* 311.0835 calc. for [C_19_H_15_ClO_2_ + H]^+^: 311.0833.

Compound **5**. To a solution of **5b** (50 mg, 0.161 mmol) and potassium iodide (27 mg, 0.161 mmol) in absolute ethanol (2 mL), dimethylamine was added (2 mL, 2 M in THF), and the mixture was refluxed for 3 days. After evaporation under reduced pressure, the crude was partitioned between water (50 mL) and ethyl acetate (50 mL), and the aqueous phase was extracted with ethyl acetate (3 × 50 mL). The combined organic phases were dried over anhydrous MgSO_4_, filtered and evaporated to dryness under reduced pressure. The product was isolated via column chromatography (silica gel, 0–10% MeOH in DCM) obtaining 45 mg of a yellowish solid (yield: 87%). TLC: Rf (10% MeOH/DCM) 0.4. ^1^H NMR (400 MHz, CDCl_3_) δ (ppm): 8.33 (d, *J* = 8.7 Hz, 1H), 7.84 (m, 4H), 7.57 (m, 5H), 7.34 (s, 1H), 4.35 (t, *J* = 5.6 Hz, 2H), 2.94 (t, *J* = 5.6 Hz, 2H), 2.43 (s, 6H). ^13^C NMR (101 MHz, CDCl_3_) δ (ppm): 197.0, 155.2, 138.2, 135.1, 133.3, 132.4, 130.2, 129.2, 128.4, 128.0, 127.9, 127.4, 125.7, 122.5, 103.9, 67.1, 58.4, 46.2. HR-ESI MS (ESI-TOF, positive mode): *m/z* 320.1645 calc. for [C_21_H_21_NO_2_ + H]^+^: 320.1645.

Compound **6**. To a solution of **5b** (40 mg, 0.129 mmol) and potassium iodide (11 mg, 0.065 mmol) in DMF (4 mL), 1-methylpiperazine (86 µL, 0.774 mmol) was added, and the mixture was stirred at 100 °C for 24 h. Then, the crude mixture was diluted with ethyl acetate (50 mL) and washed with water (3 × 50 mL). The organic phase was dried over anhydrous MgSO_4_, filtered and evaporated to dryness under reduced pressure. The product was isolated via column chromatography (silica gel, 0–10% MeOH in DCM) obtaining 30 mg of an orange solid (yield: 63%). TLC: Rf (10% MeOH in DCM) 0.5. ^1^H NMR (400 MHz, CDCl_3_) δ (ppm): 8.30 (d, *J* = 8.8 Hz, 1H), 7.84 (m, 3H), 7.80 (s, 1H), 7.55 (m, 5H), 7.33 (d, *J* = 1.3 Hz, 1H), 4.37 (t, *J* = 5.7 Hz, 2H), 3.00 (t, *J* = 5.7 Hz, 2H), 2.73 (s, 4H), 2.52 (s, 4H), 2.31 (s, 3H). ^13^C NMR (101 MHz, CDCl_3_) δ (ppm): 196.8, 155.0, 138.2, 135.1, 133.3, 132.3, 130.1, 129.2, 128.4, 127.90, 127.4, 125.6, 122.4, 104.0, 67.0, 57.2, 55.2, 53.6, 46.0. HR-ESI MS (ESI-TOF, positive mode): *m*/*z* 375.2068 calc. for [C_24_H_26_N_2_O_2_ + H]^+^: 375.2069.

### 4.3. Fluorescence Binding Experiments

These experiments measured compound association to an RRE subdomain IIB RNA oligonucleotide labeled with fluorescein at extrahelical loop nucleotide U23 (Figure 1) and were carried out in a Spectramax iD5 microplate reader (Molecular Devices) using 100 nM RNA dissolved in 10 mM sodium phosphate, pH 6.6, and 0.1 mM EDTA, as described before [9,13,17]. The equilibrium dissociation constants, K_d_, were determined by fitting the fluorescence intensity curves to a two-state binding model [18] with GraphPad Prism software. With the exception of **2**, no compound was found to fluoresce in the conditions of the assay, and compound fluorescence was subtracted from the signal obtained in the presence of RNA. All fluorescence experiments were carried out at least three times for each compound.

### 4.4. EMSA

These assays were used to monitor full-length RRE–Rev inhibition and involved the viral protein Rev and a 234 nt RRE sequence (Figure 1), obtained as reported previously [9]. RRE was prepared in native conditions using a procedure based on electrophoresis. The reactions used 70 nM RRE and 0.75 μM Rev dissolved in an aqueous solution containing 10 mM HEPES, pH 7.5, 300 mM KCl, 1 mM MgCl_2_ and 0.5 mM EDTA, and the samples were loaded onto 8% polyacrylamide gels with TB running buffer. Gels were run at 4 °C for 3 h at 150 V, and the bands were stained with SYBR Gold and quantified with ImageJ software. In all cases, we monitored the disappearance of the bands corresponding to low-order RRE–Rev complexes (Figure 4A) using a sigmoidal inhibitory model and GraphPad Prism. At least three independent experiments were conducted for each compound.

### 4.5. Antiretroviral Activity and Cellular Toxicity

The methodology used to perform these experiments in MT-2 cell culture was reported previously [6,9,13,17]. Infectious HIV-1_NL4-3-Ren_ supernatants were obtained from transfection in 293T cells with plasmid pNL4-3-Ren, where a Renilla luciferase reporter gene replaces the *nef* gene of the wild-type pNL4-3 HIV-1 clone [19,20], which includes LTR promoters. MT-2 cells were infected with these supernatants (100,000 RLUs/well) in the presence of the compounds, and anti-HIV activity was quantified 48 h post-infection by determining Renilla luciferase activity in cell lysates compared to a non-treated control (100%), following the manufacturer’s instructions (Renilla Assay System, Promega). Briefly, 100 µL of Renilla Assay System buffer was added to obtain cell lysates, and 35 µL of these lysates was then transferred to a white 96-well microplate. RLUs were recorded after injecting 35 µL of Renilla substrate (10 s reading) using a luminometer (Berthold Detection Systems). Cellular viability was evaluated in mock-infected cells similarly treated with the same concentrations of compounds using the CellTiter-Glo assay (Promega). To assess the antiretroviral activity of benfluron in human primary cells, PBMCs were isolated from healthy donors via Ficoll density gradient centrifugation and subsequently cultured in a PHA (1 µg/mL) and IL-2 (100 U/mL)-enriched medium for 48 h at 37 °C. Activated PBMCs were infected with HIV-1_NL4-3_ [20] at a multiplicity of infection of 0.001 and cultured in the presence of serial dilutions (5 nM–100 µM) of benfluron. Three days post-infection, the supernatant was collected, and the HIV-inhibitory effect of the drug was measured using the Alliance HIV-1 p24 Antigen Elisa Kit (Perkin Elmer). The toxicity of the compound in PBMCs was evaluated as explained above for MT-2 cells. In both experiments, the potential background toxicity and antiviral effect of the DMSO vehicle used to solubilize the molecule were determined in parallel. In all cases, EC_50_ and CC_50_ values were calculated with GraphPad Prism using log(inhibitor) vs. response non-linear curve fitting. All cellular assays were performed in triplicate.

## Figures and Tables

**Table 1:** RRE subdomain IIB recognition and full-length RRE–Rev inhibition data of benfluron and benfluron analogs **1**–**6**.

Compound ^a^	K_d_ (RRE IIB) (µM)	IC_50_ (RRE–Rev) (µM)
benfluron	4.83 (4.39–5.31, 0.9779) ^b^	5.0 (3.4–7.5, 0.8340)
**1**	45.6 (39.8–52.5, 0.9863)	>50
**2**	>100	>50
**3**	106 (64.1–197, 0.9007)	>50
**4**	77.5 (55.0–113.1, 0.9591)	>50
**5**	95.1 (72.3–128, 0.9835)	29 (20–43, 0.8137)
**6**	34.0 (28.6–40.6, 0.9662)	45 (27–87, 0.5991)

^a^ RRE subdomain IIB K_d_ values were obtained with fluorescence experiments involving an RRE IIB hairpin labeled with fluorescein at position U23, and full-length RRE–Rev IC_50_ values were measured from low-order RRE–Rev complex EMSA bands; 95% confidence intervals and R^2^ fit coefficients are shown in parentheses when applicable. ^b^ For a comparison, we previously obtained a K_d_ of 1.39 µM from fluorescence assays involving a RRE IIB hairpin labeled with 2-aminopurine at position A19 [13].

**Table 2:** Results of MT-2 cellular assays for benfluron analogs. Inhibitory activity (EC_50_) in experiments based on HIV-1 infection and cellular toxicity (CC_50_).

Compound	EC_50_ (µM)	CC_50_ (µM)
Benfluron	0.830 (0.580–1.17, 0.9707) ^b^	28.1 (12.0–65.7, 0.8513) ^b^
**1**	>100	>100
**2**	~100	~100
**3**	~100	~100
**4**	17.0 (10.0–29.1, 0.8180)	28.7 (16.3–50.8, 0.7588)
**5**	19.4 (11.5–32.9, 0.8178)	49.7 (23.4–113, 0.5291)
**6**	24.2 (16.2–36.3, 0.7632)	>20 <100

95% confidence intervals and R^2^ fit coefficients are shown in parentheses when applicable. ^b^ Data from [13].

## Data Availability

Data are contained in the article and Supplementary Materials or will be provided by the authors.

## Supplementary Materials

The following supporting information can be downloaded at: https://www.mdpi.com/article/10.3390/molecules28207031/s1, Figure S1: Antiretroviral activity and cellular toxicity of benfluron in PBMCs; Figures S2–S30: ^1^H and ^13^C NMR, HR ESI-MS and reversed-phase HPLC data of compounds.

## Sample Availability

Not available.
