# Supplementary material for: Exploring the HIV-1 Rev Recognition Element (RRE)–Rev Inhibitory Capacity and Antiretroviral Action of Benfluron Analogs
Source: Molecules. 2023 Oct 11;28(20):7031. doi: 10.3390/molecules28207031 (PMC10609163; doi:10.3390/molecules28207031)
Supplement: Supplementary file 1 [file molecules-28-07031-s001.zip › molecules-2601149-supplementary.pdf]

Exploring the HIV-1 Rev Recognition Element (RRE)-Rev inhibitory capacity and antiretroviral action of benfluron analogs

Supplementary Materials

Sergi Chumillas, Saurabh Loharch, Manuela Beltrán, Mateusz P. Szewczyk, Silvia Bernal, Maria C. Puertas, Javier Martinez-Picado, José Alcamí, Luis M. Bedoya, Vicente Marchán*, José Gallego*

Supplementary Figures

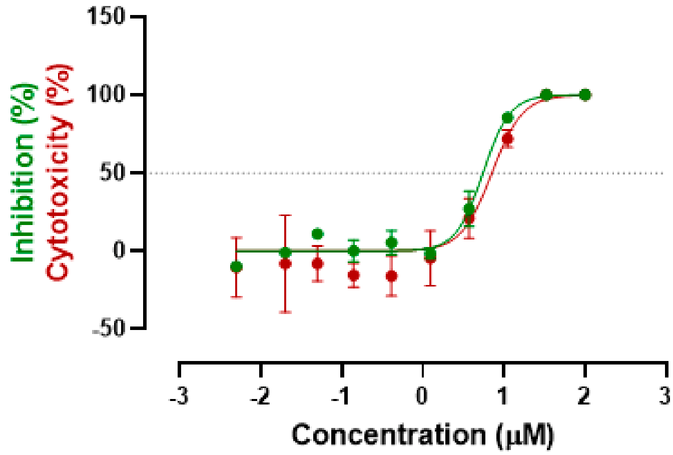

Figure S1. Antiretroviral activity and cellular toxicity of benfluron in PBMC. In this cell type this agent had an antiretroviral EC_{50} of 5.50 μM (95% confidence interval 4.65-6.54 μM, R^2=0.9781) and a toxic concentration CC_{50} of 7.09 μM (4.81-10.3 μM, R^2=0.9147), making for a selectivity index of 1.3.

^{1}H and ^{13}C NMR spectra and HR ESI-MS of benfluron analogs 2-6

Compound 2

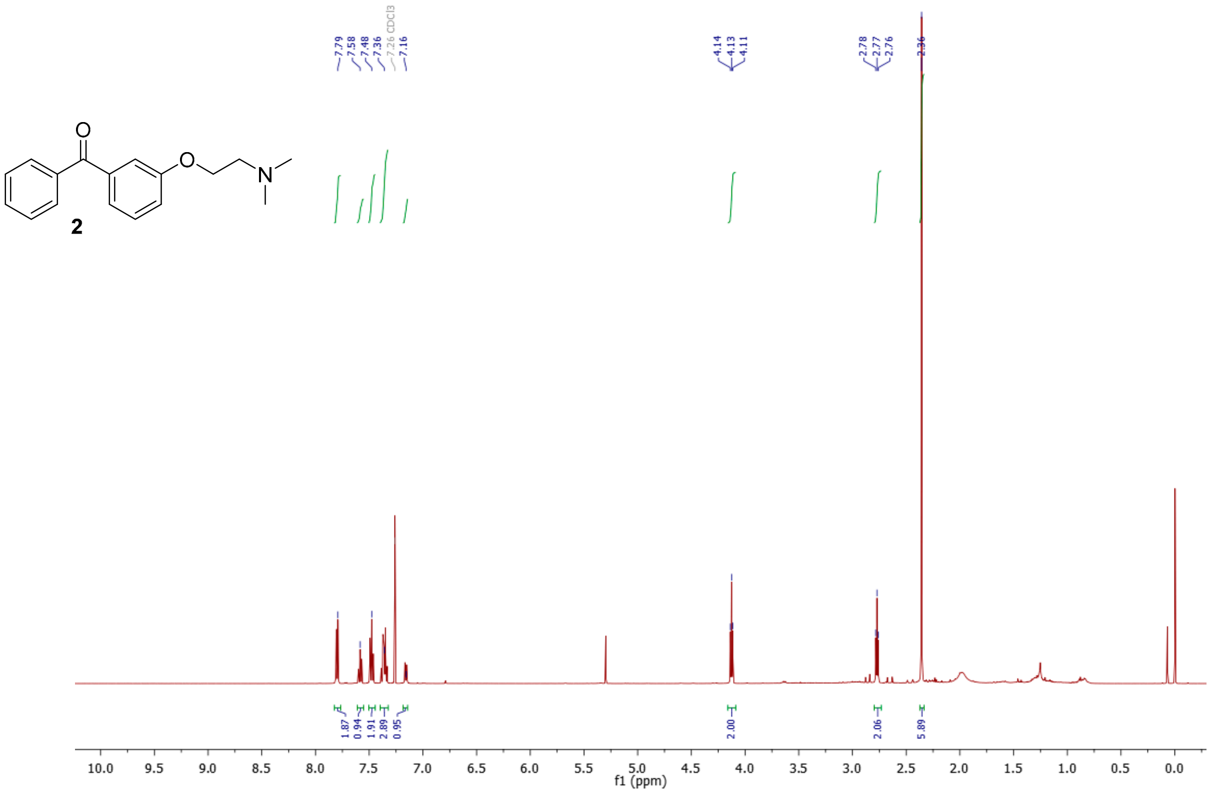

2

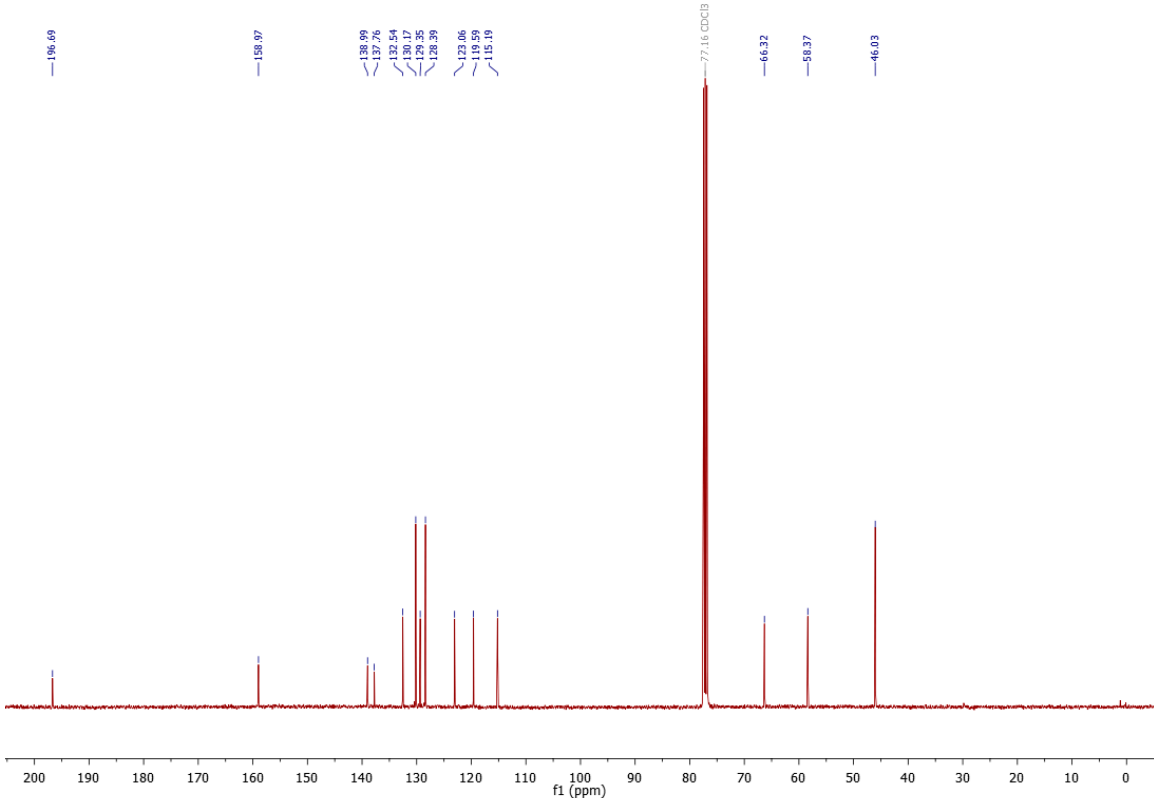

Figure S2. ^{1}H (400 MHz) and ^{13}C NMR (101 MHz) spectra of compound **2** in $CDCl_3$.

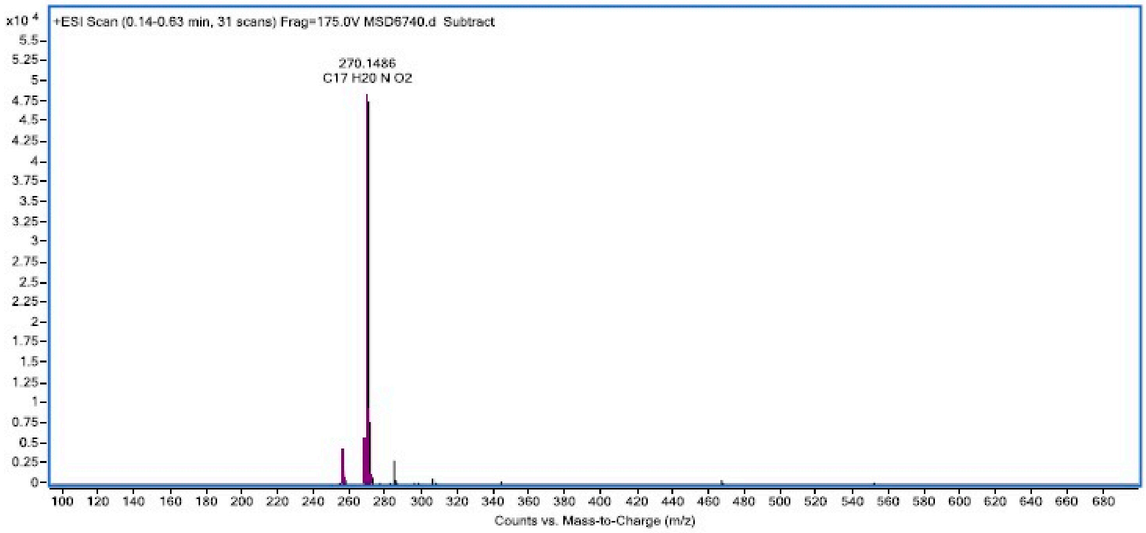

Figure S3. HR ESI-MS spectrum of compound **2** in MeOH.

Compound 3

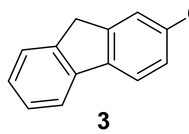

3

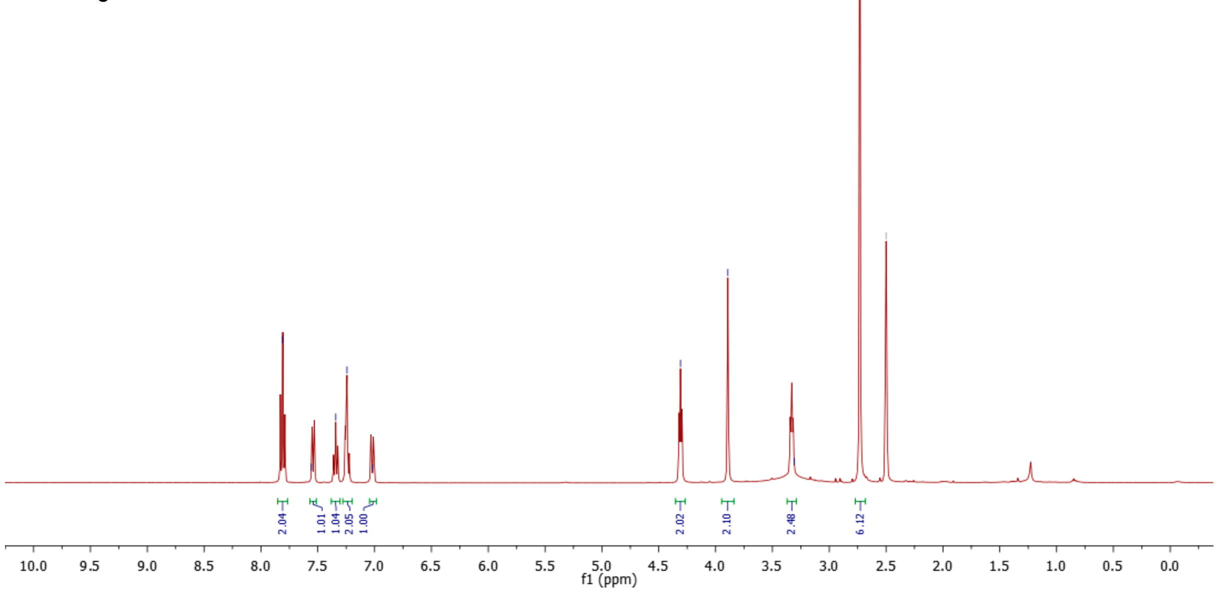

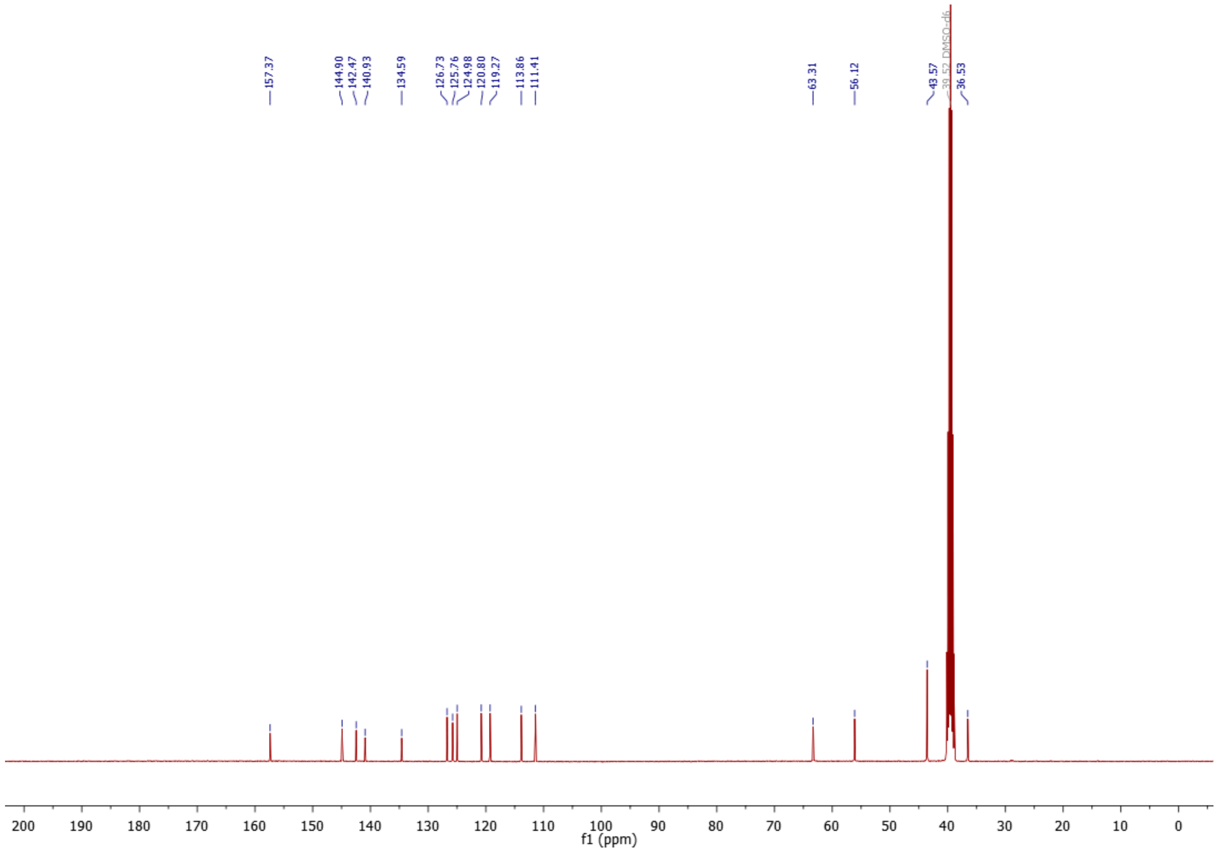

Figure S4. ^{1}H (400 MHz) and ^{13}C NMR (101 MHz) spectra of compound **3** in DMSO-*d*$_6$.

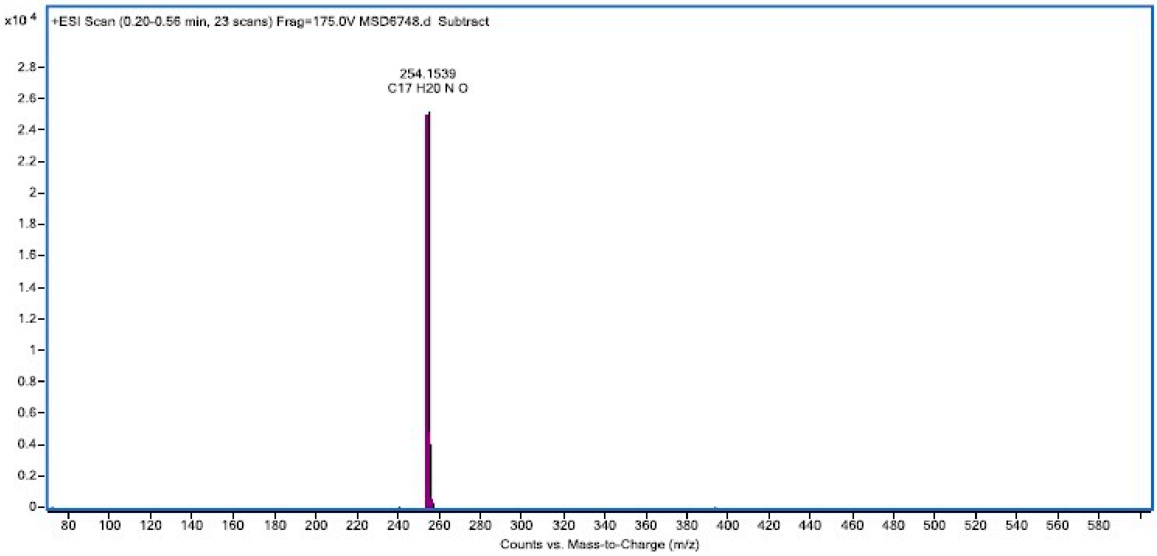

Figure S5. HR ESI-MS spectrum of compound **3** in MeOH.

Compound 4

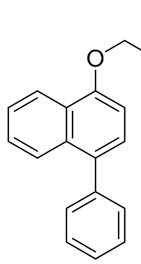

4

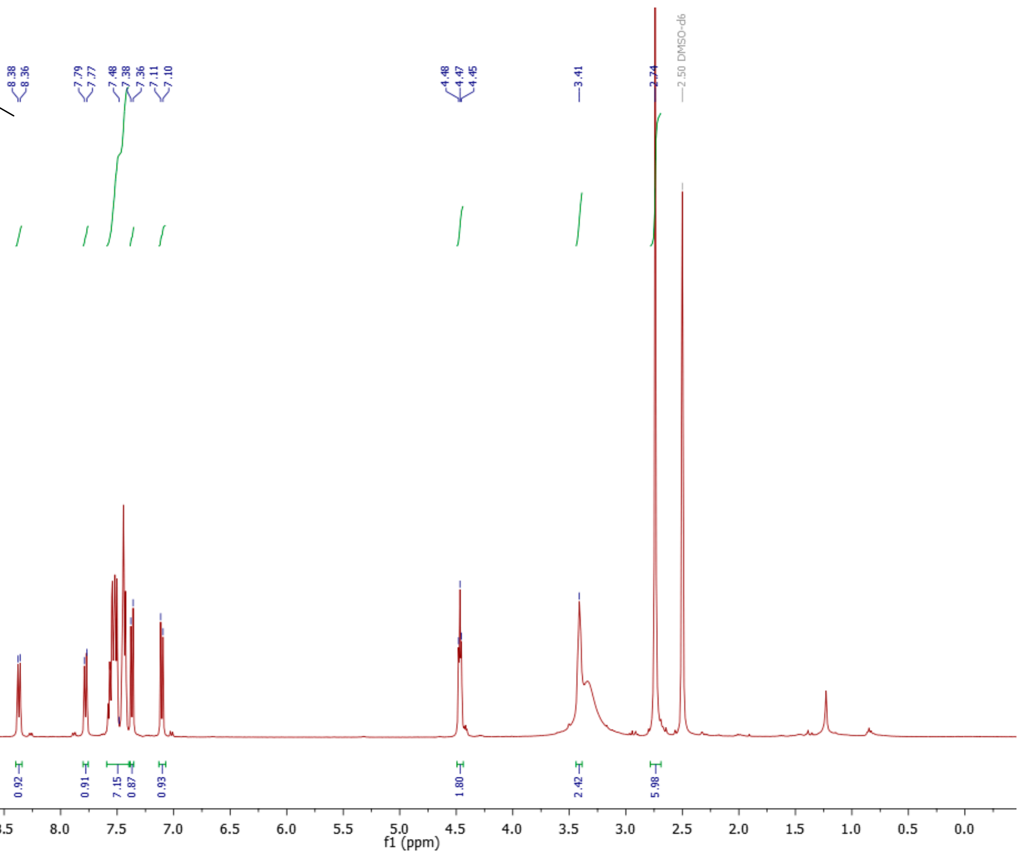

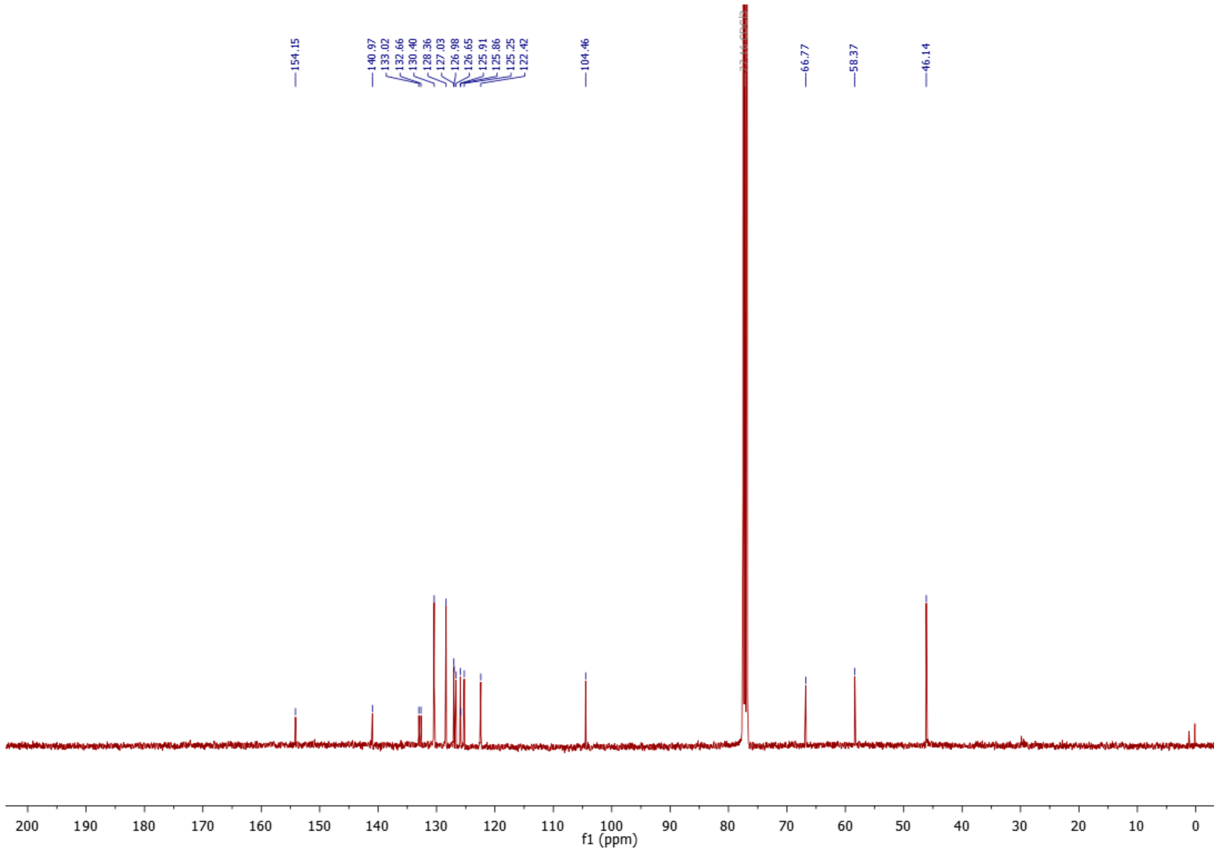

Figure S6. ¹H (DMSO-d_6, 400 MHz) and ¹³C NMR ($CDCl_3$, 101 MHz) spectra of **4**.

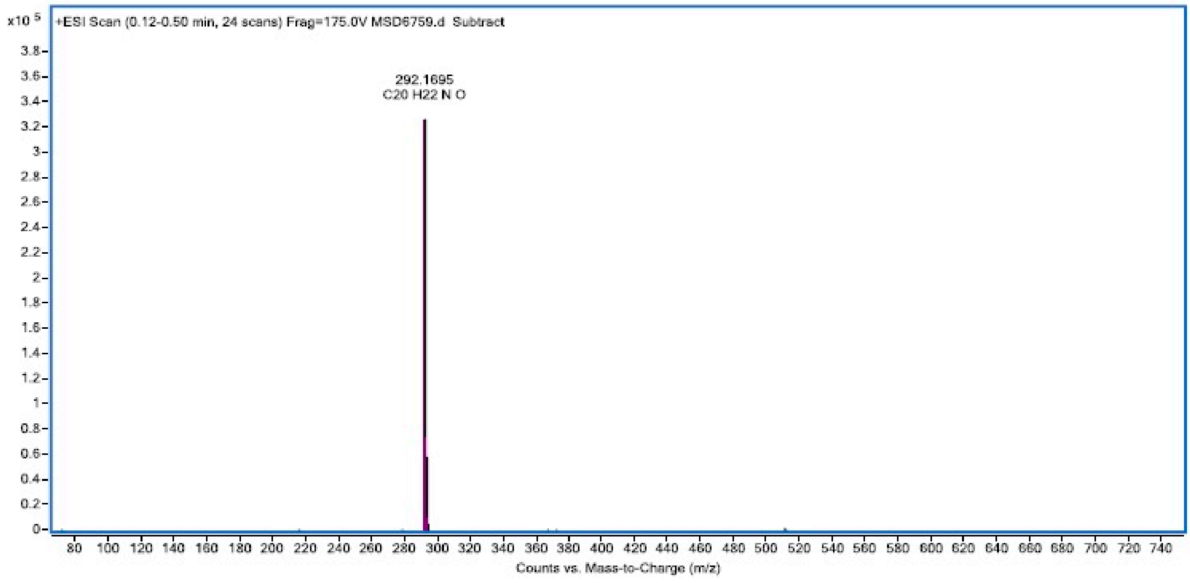

Figure S7. HR ESI-MS spectrum of compound **4** in MeOH.

Compound 5

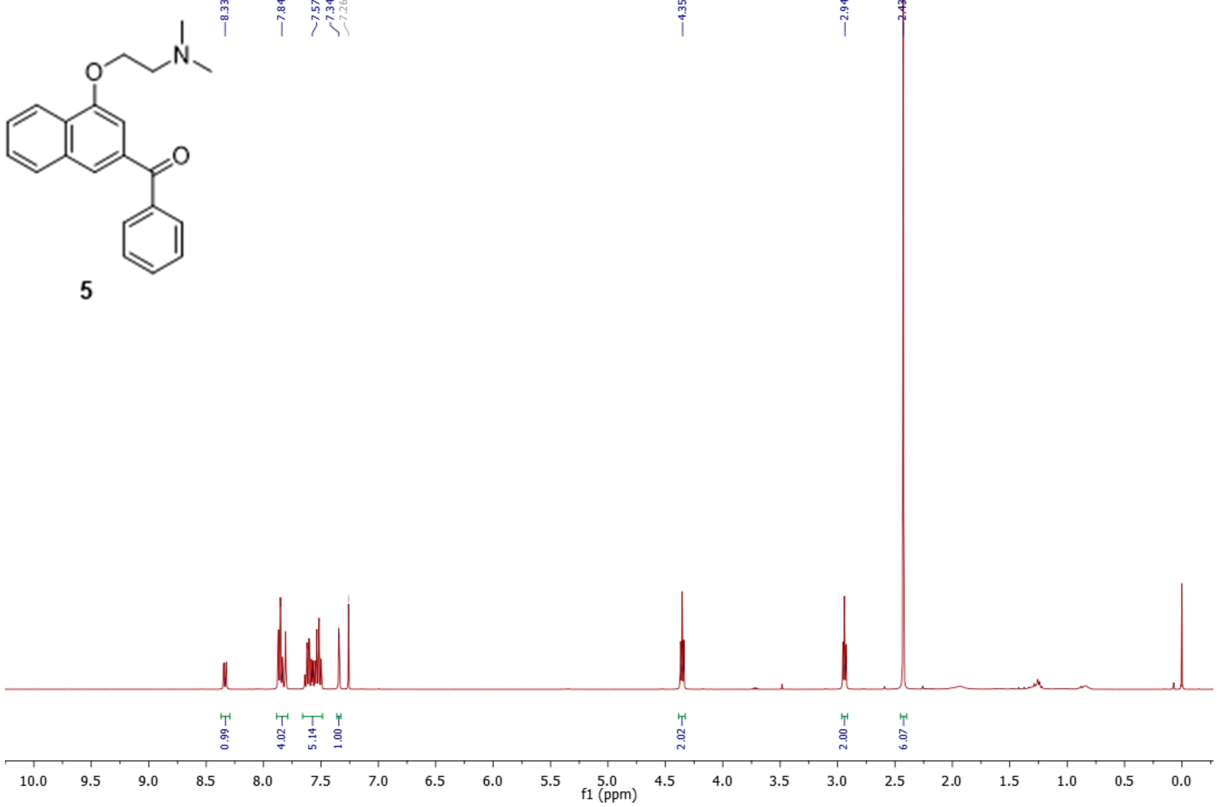

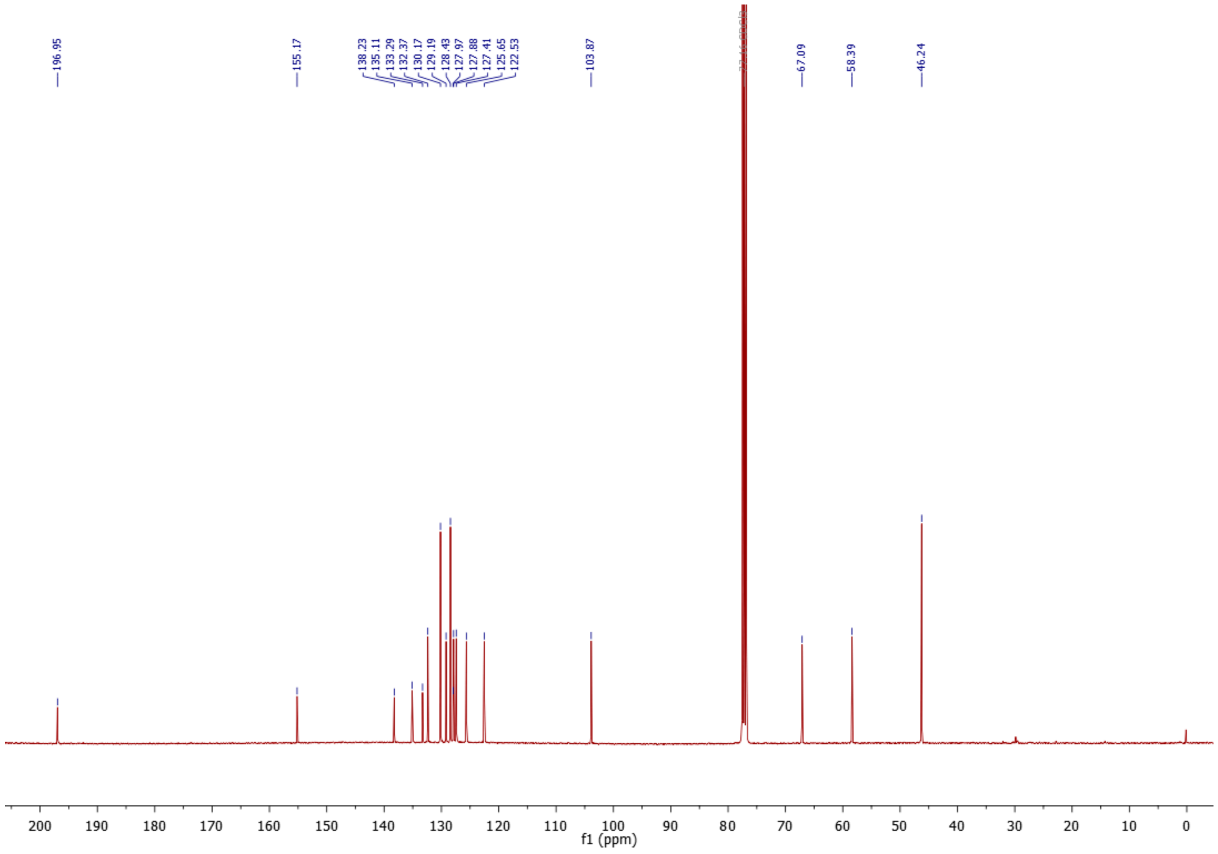

Figure S8. ^{1}H (400 MHz) and ^{13}C NMR (101 MHz) spectra of compound **5** in $CDCl_3$.

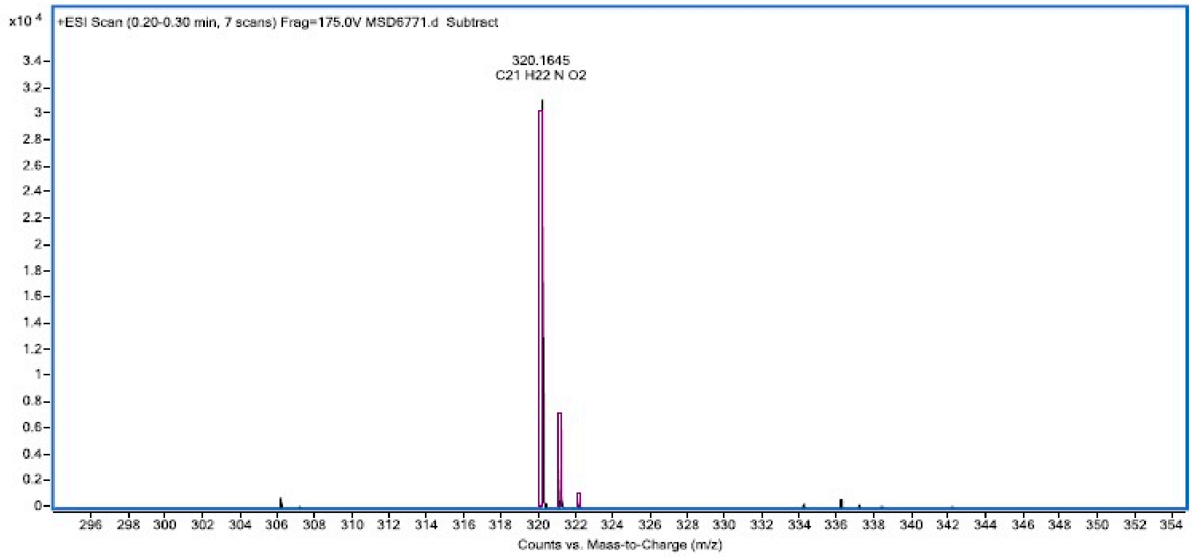

Figure S9. HR ESI-MS spectrum of compound **5** in MeOH.

Compound 6

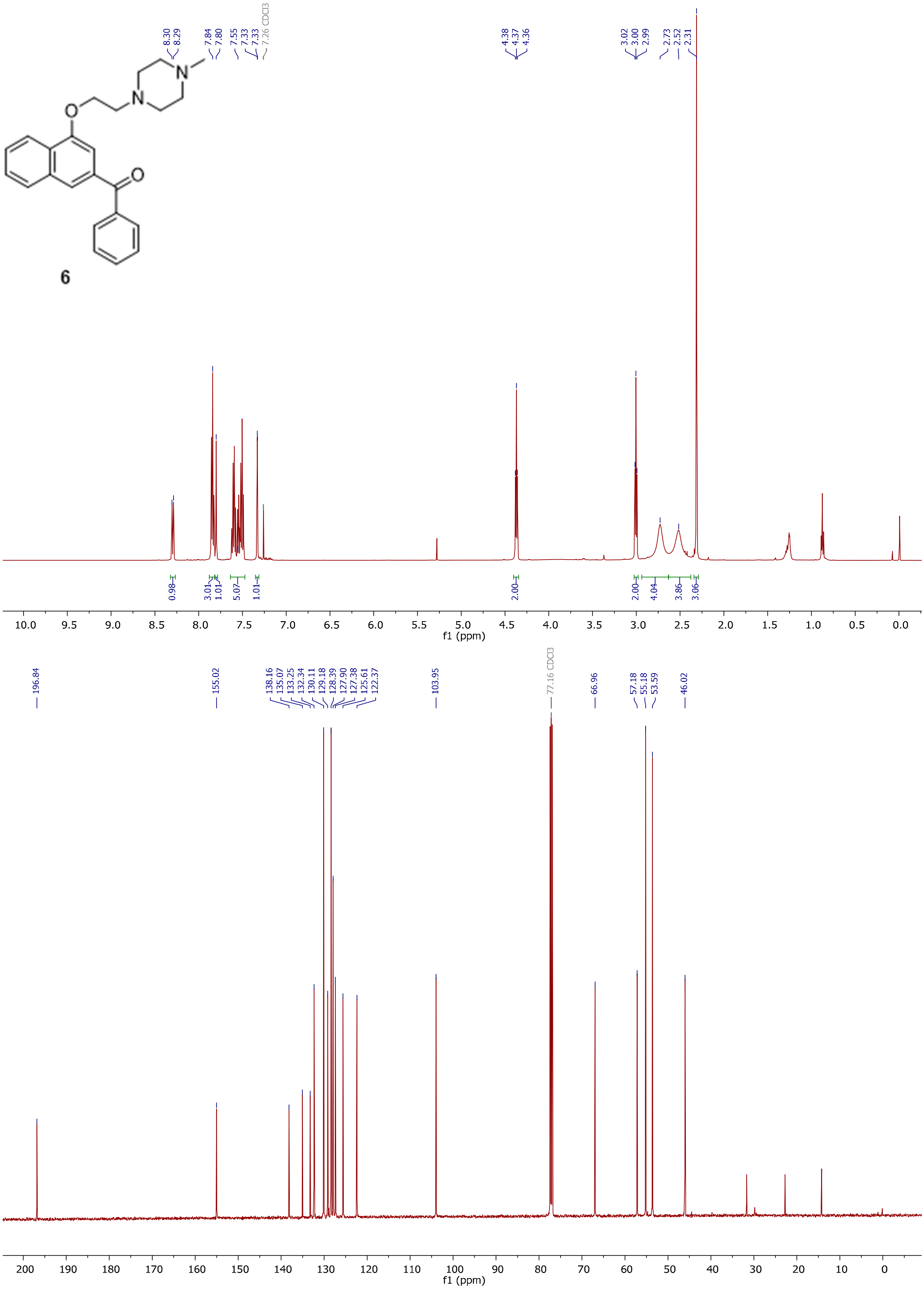

Figure S10. ^{1}H (400 MHz) and ^{13}C NMR (101 MHz) spectra of compound **6** in $CDCl_3$.

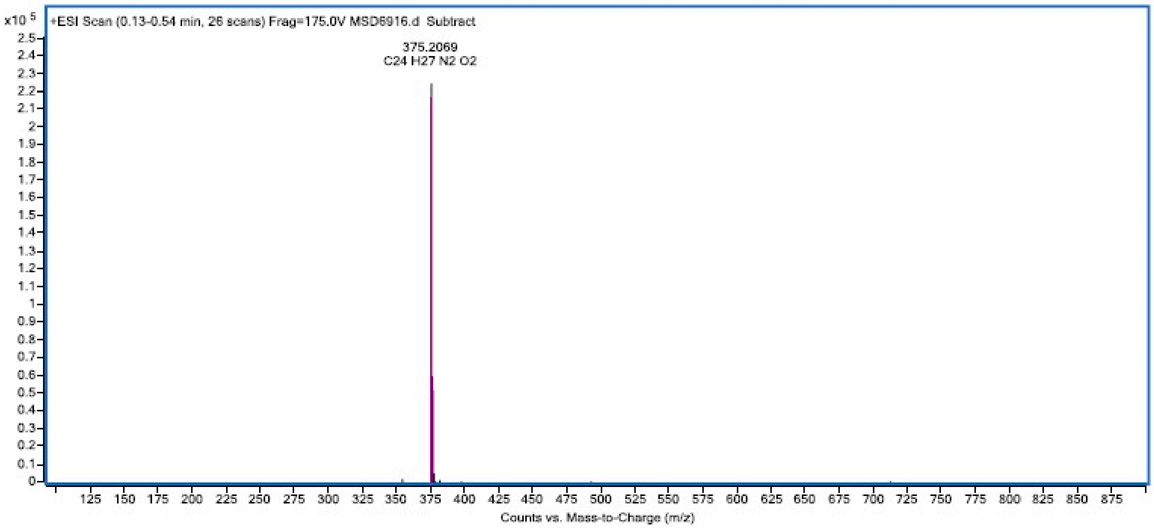

Figure S11. HR ESI-MS spectrum of compound **6** in MeOH.

Reversed-phase HPLC analysis of benfluron analogs (2-6):

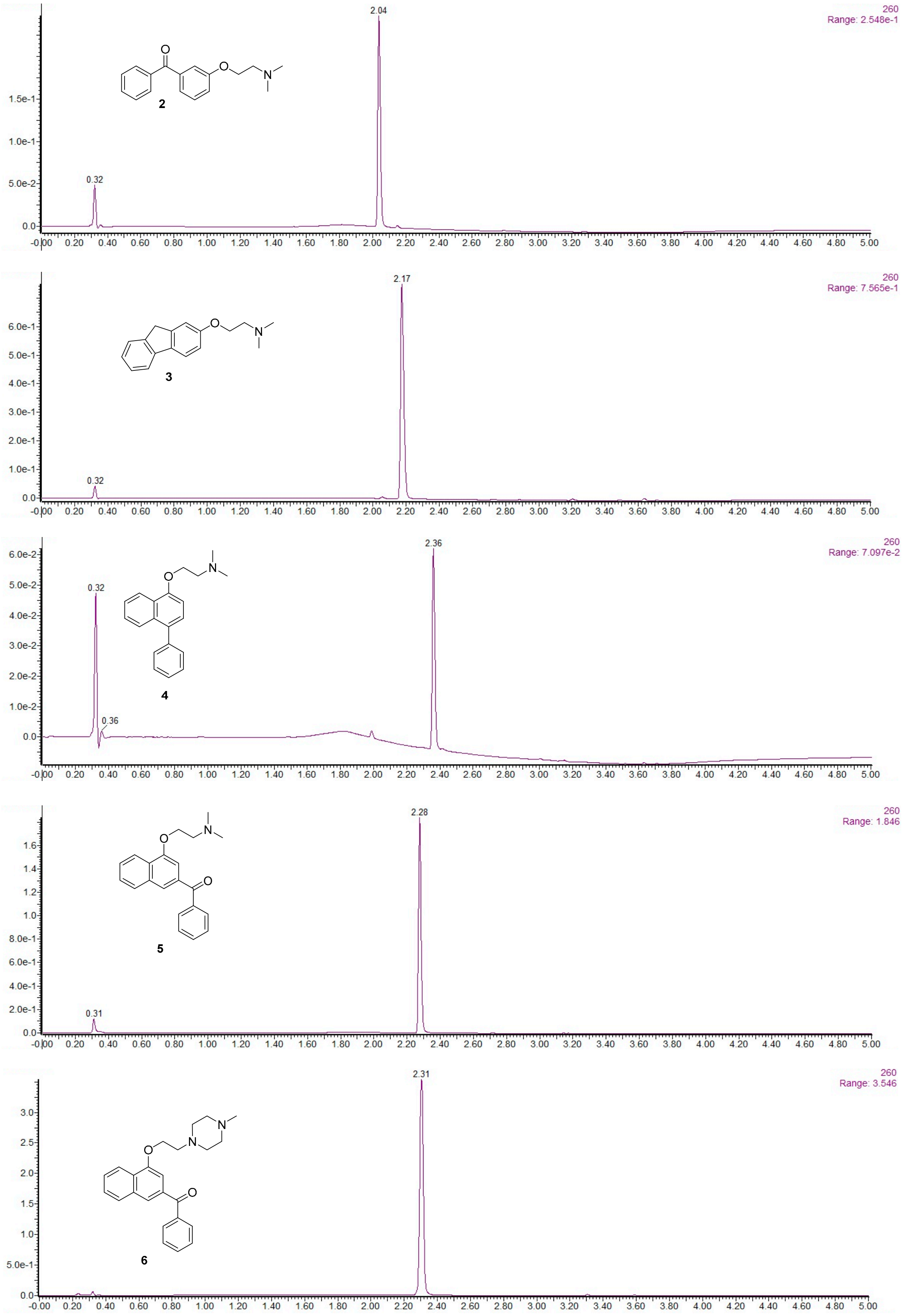

Figure S12. Reversed-phase HPLC chromatograms of benfluron analogs **2-6**.

^{1}H and ^{13}C NMR spectra and HR ESI-MS of compounds 2b-5b

Compound 2b

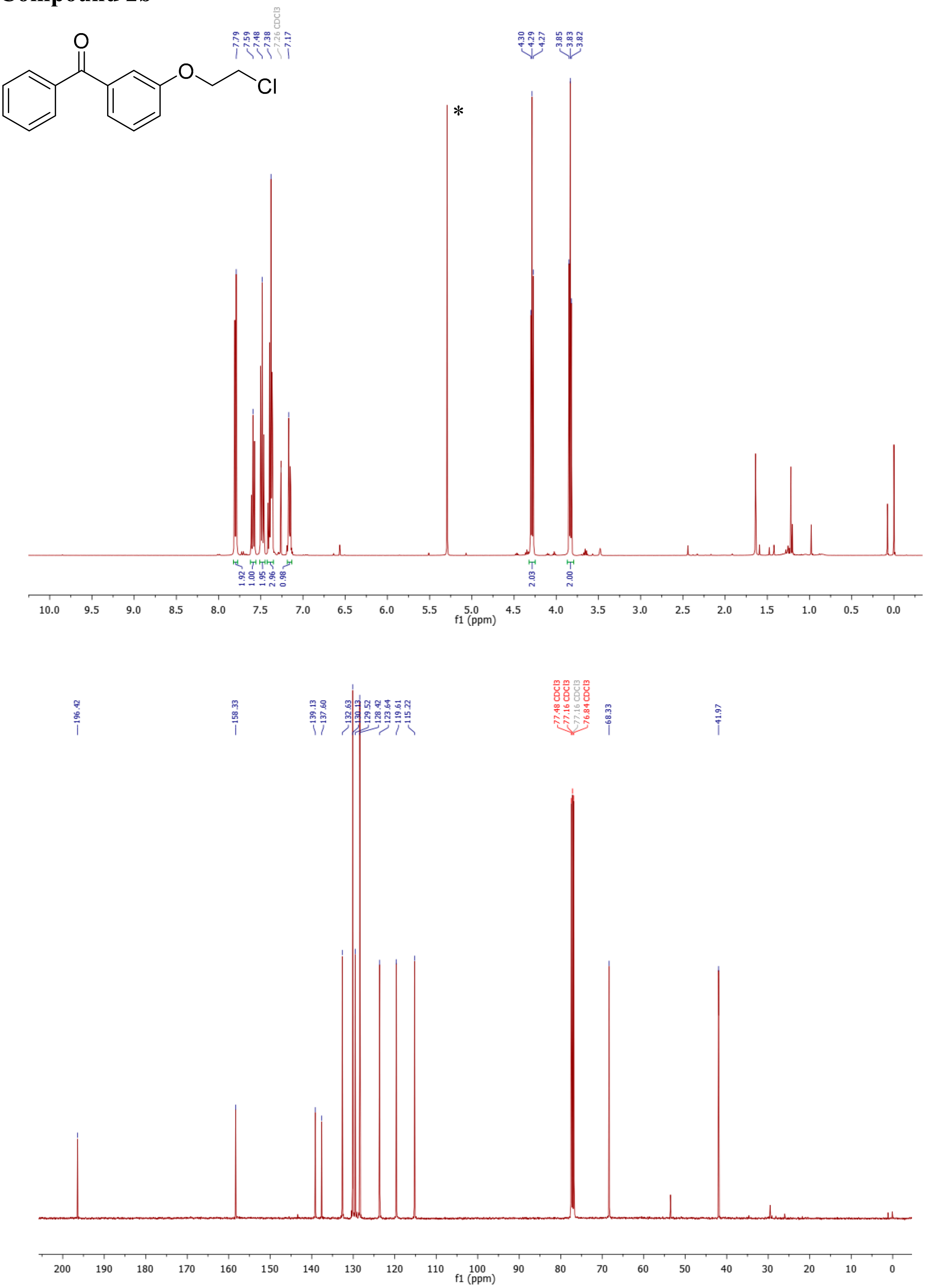

Figure S13. ^{1}H (400 MHz) and ^{13}C NMR (101 MHz) spectra of compound **2b** in $CDCl_3$.

* DCM residue signal.

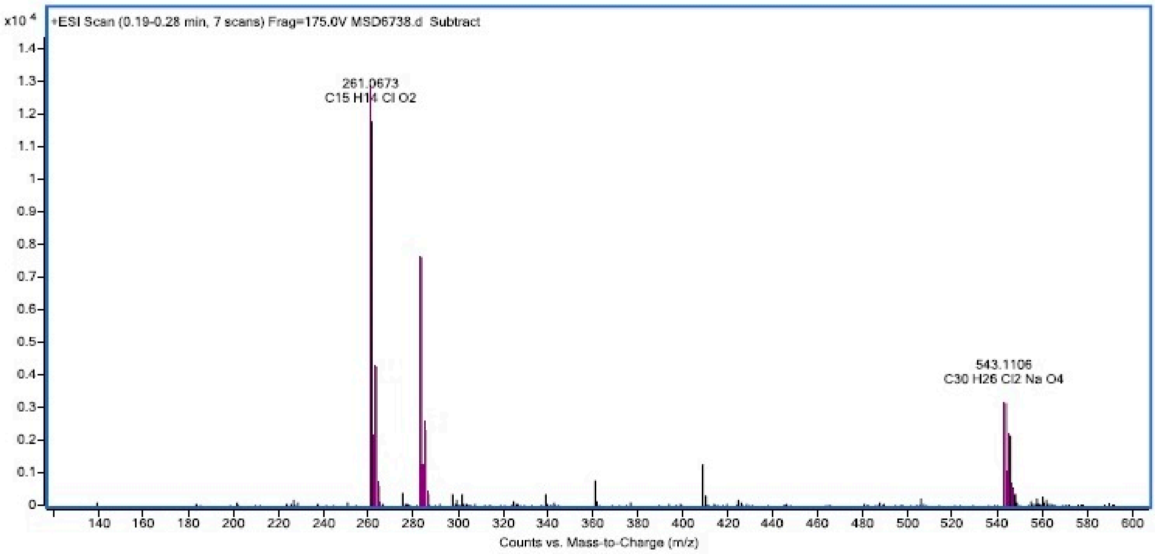

Figure S14. HR ESI-MS spectrum of compound **2b** in MeOH.

Compound 3b

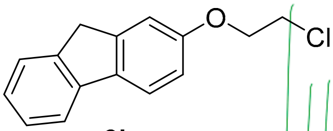

3b

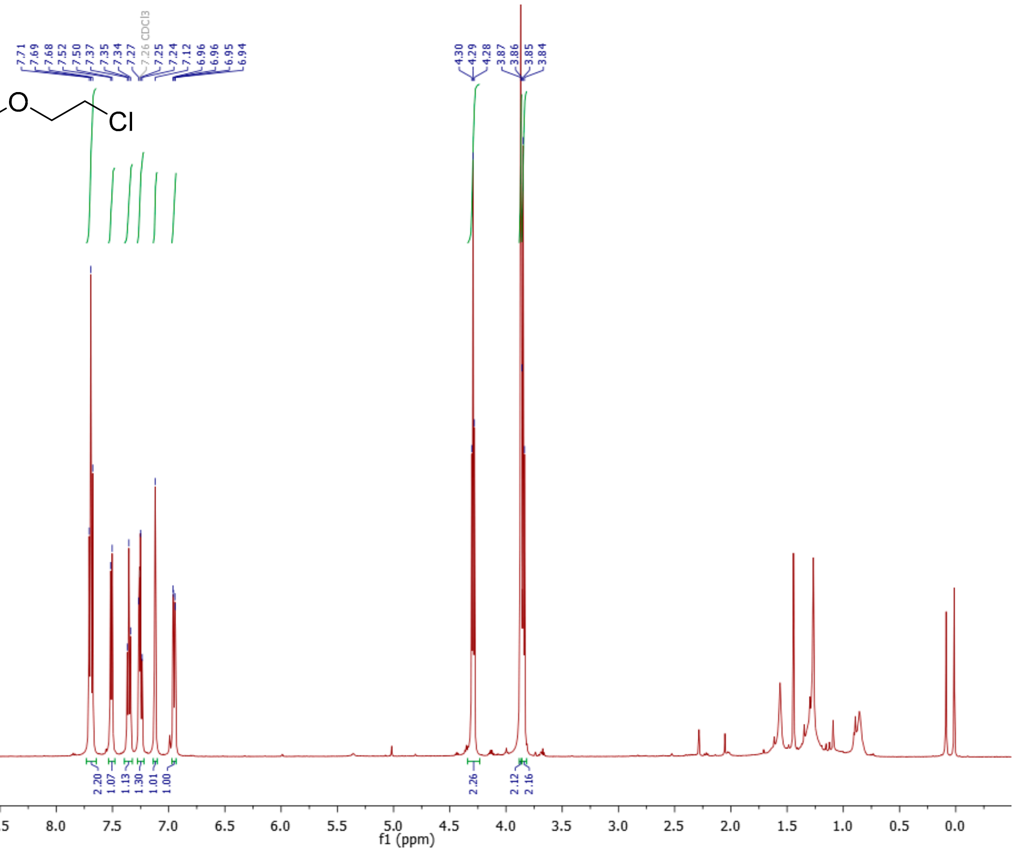

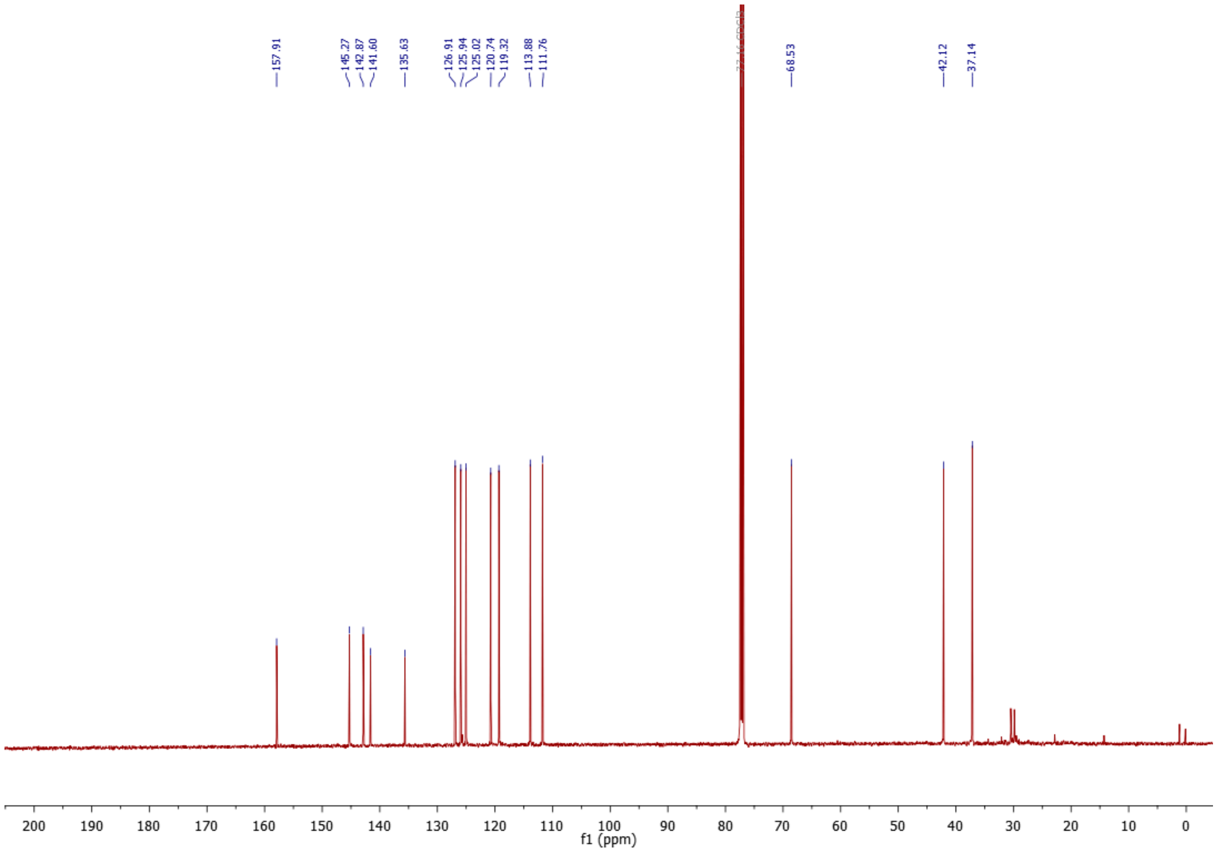

Figure S15. ^{1}H (400 MHz) and ^{13}C NMR (101 MHz) spectra of compound **3b** in $CDCl_3$.

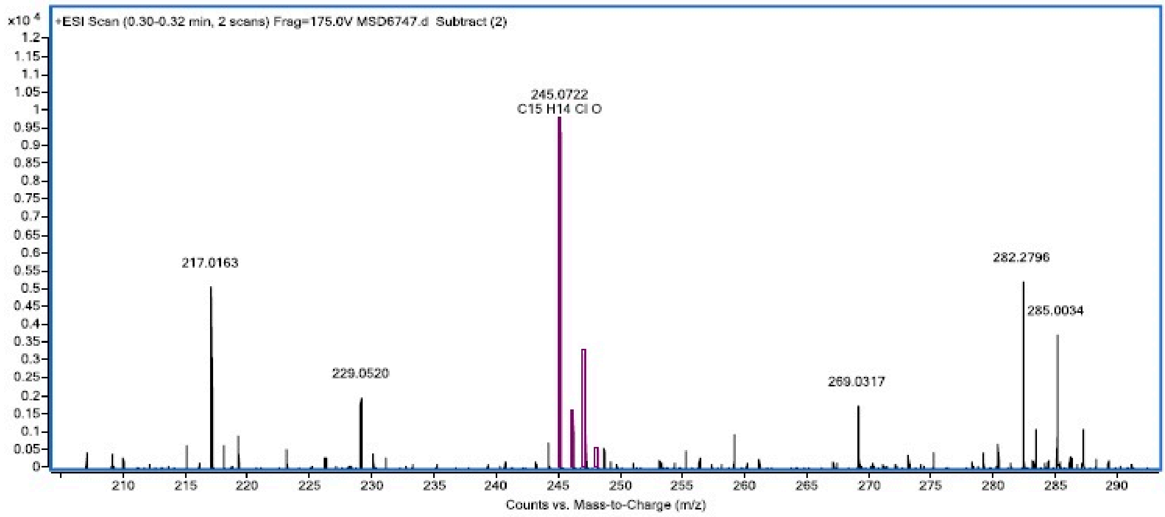

Figure S16. HR ESI-MS spectrum of compound **3b** in MeOH.

Compound 4b

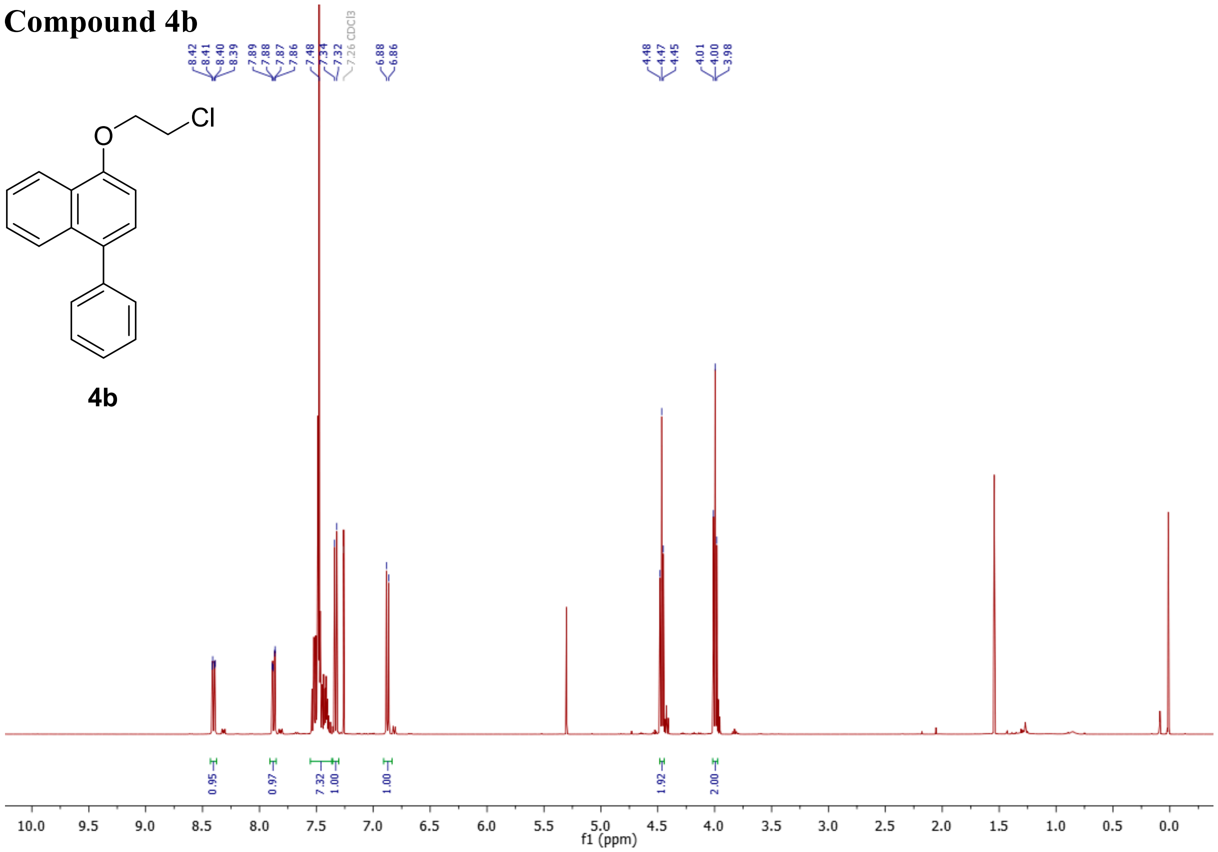

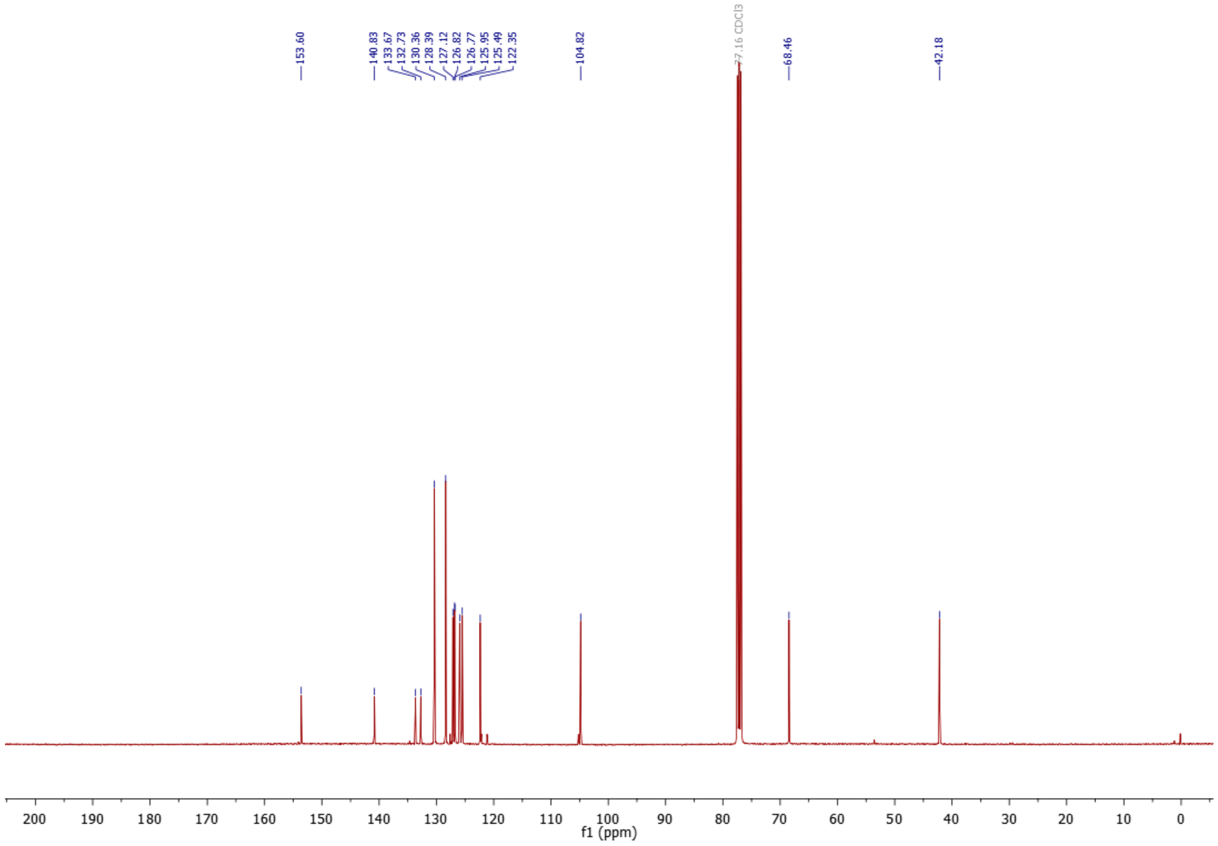

Figure S17. ^{1}H (400 MHz) and ^{13}C NMR (101 MHz) spectra of compound **4b** in $CDCl_3$.

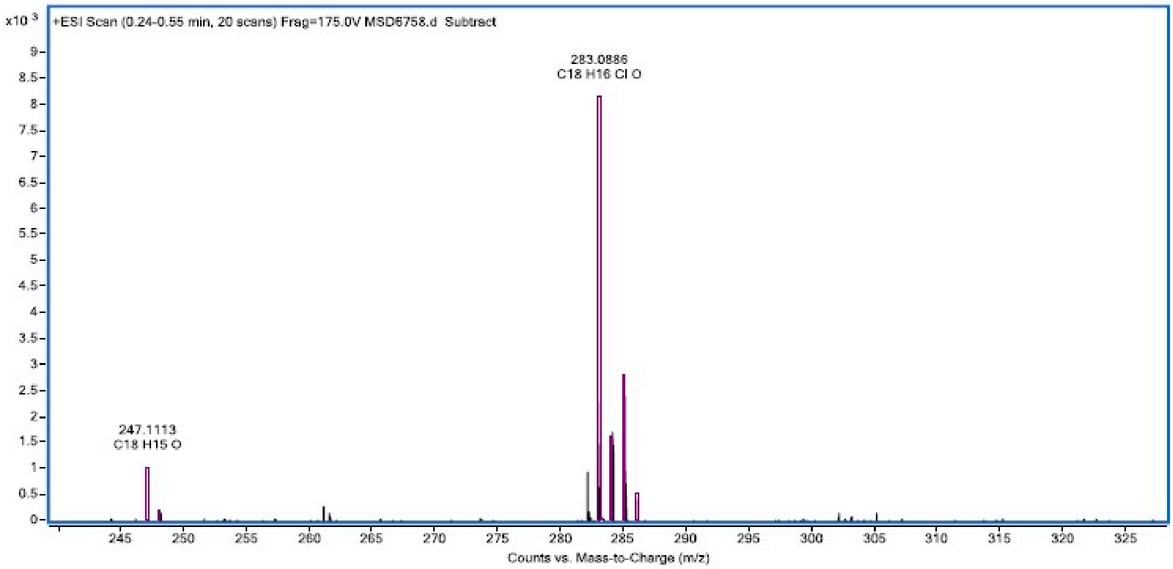

Figure S18. HR ESI-MS spectrum of compound **4b** in MeOH.

Compound 5b

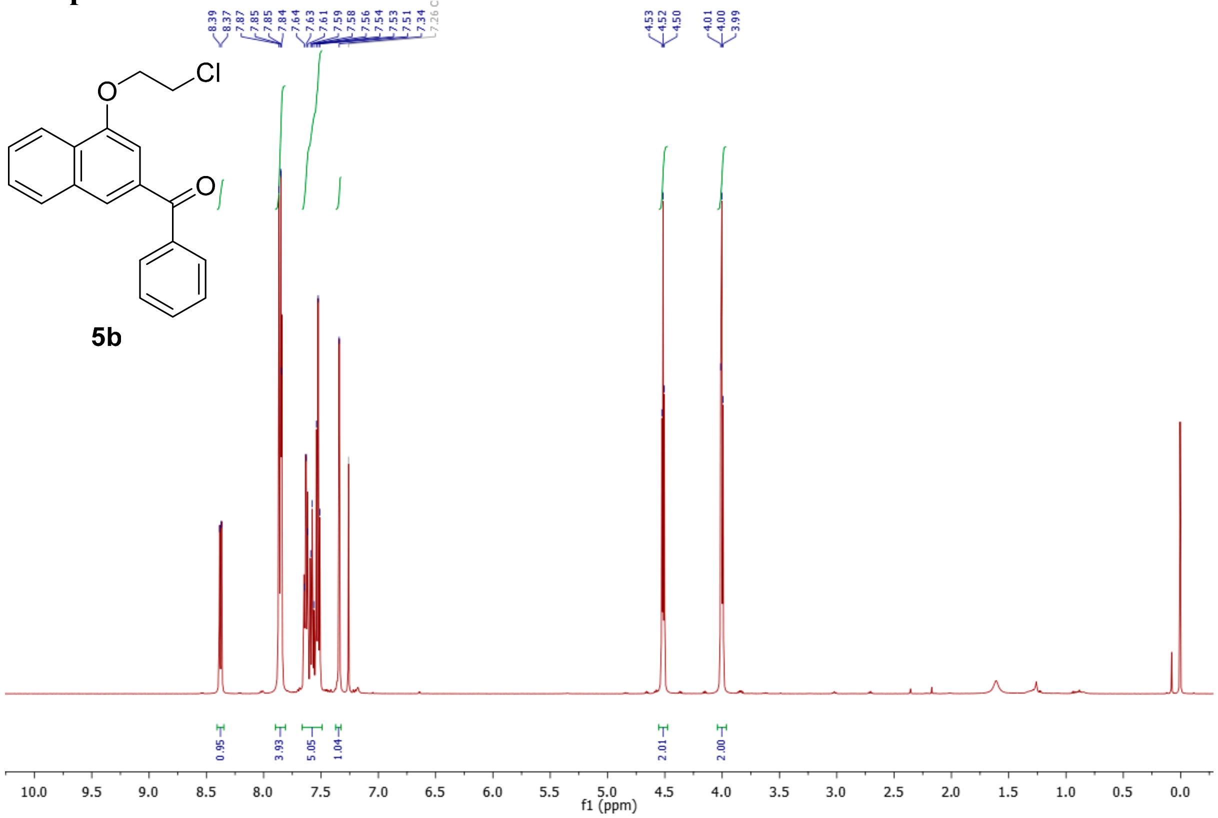

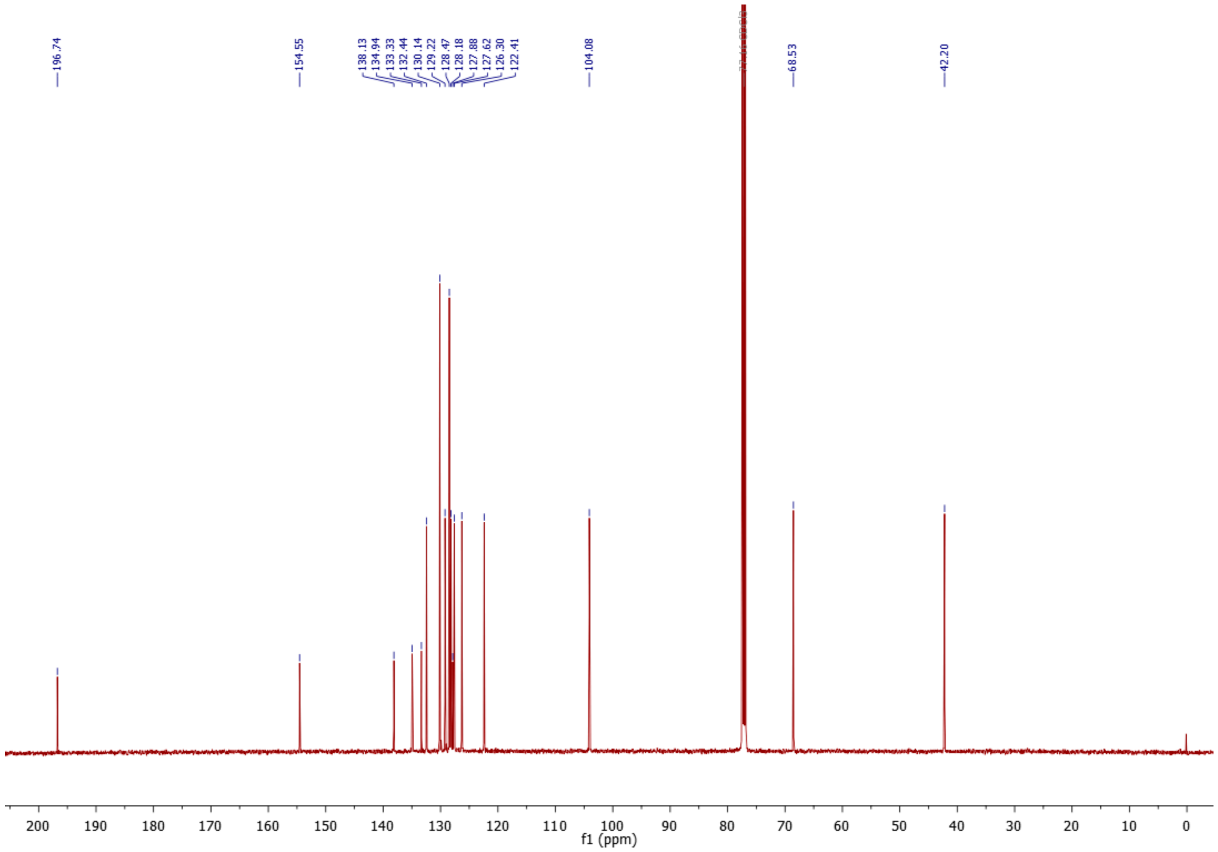

Figure S19. ^{1}H (400 MHz) and ^{13}C NMR (101 MHz) spectra of compound **5b** in $CDCl_3$.

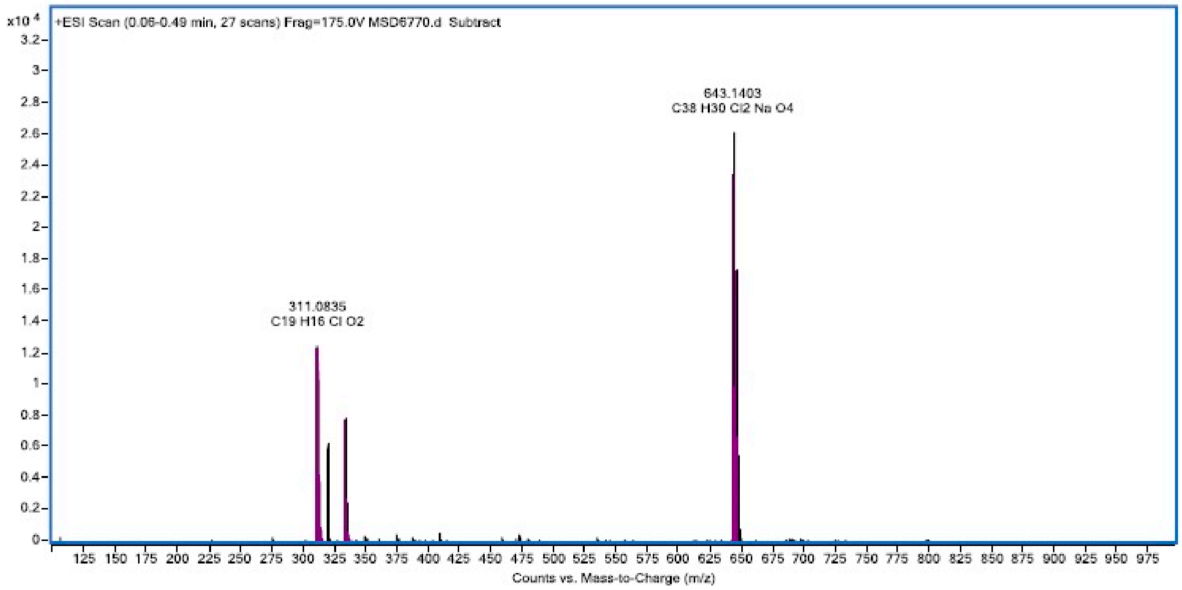

Figure S20. HR ESI-MS spectrum of compound **5b** in MeOH.

Reversed-phase HPLC analysis of compounds 2b-5b

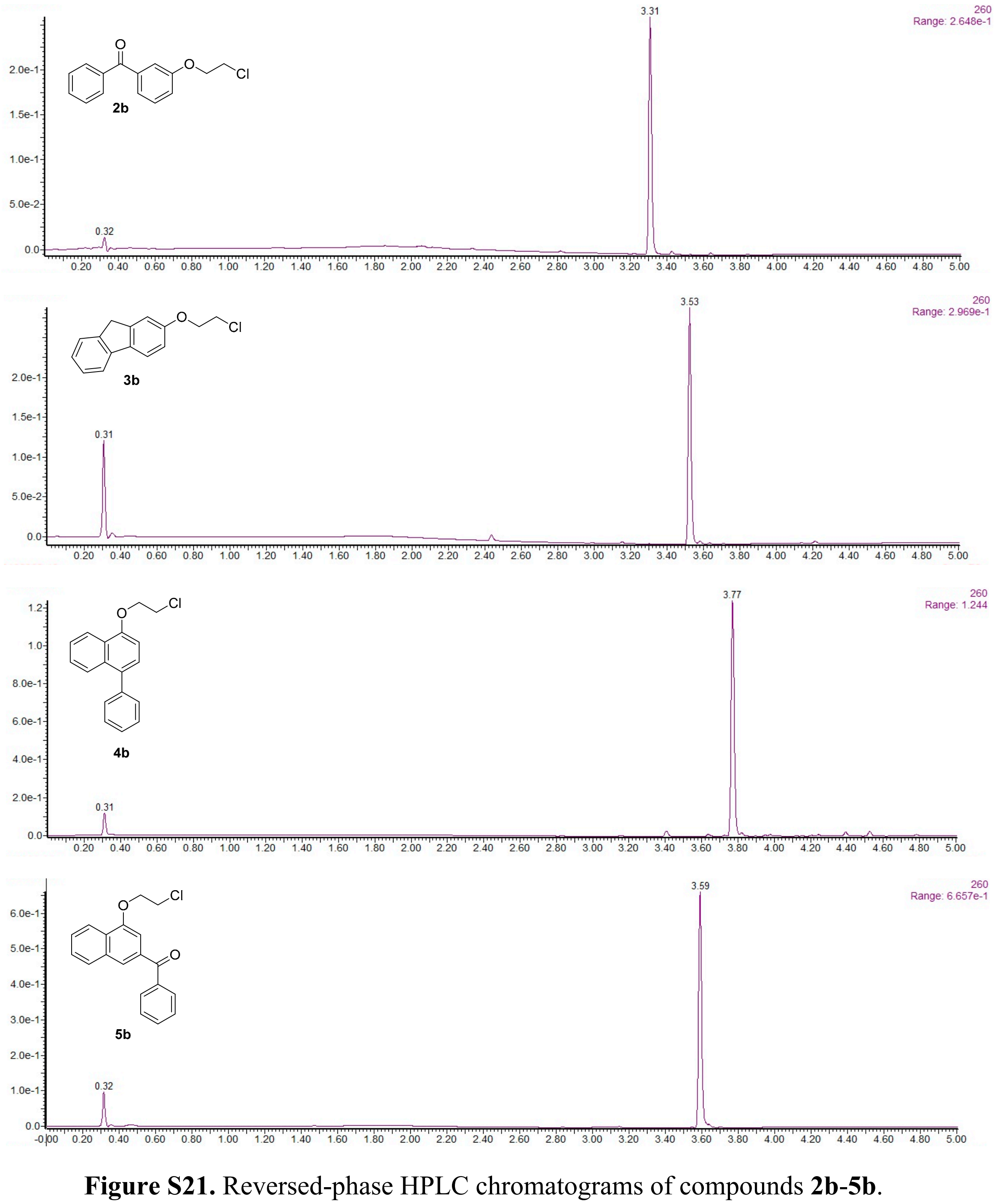

Figure S21. Reversed-phase HPLC chromatograms of compounds **2b-5b**.

^{1}H and ^{13}C NMR spectra, HR ESI-MS and HPLC analysis of compounds 4a-5a

Compound 4a

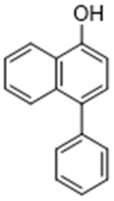

4a

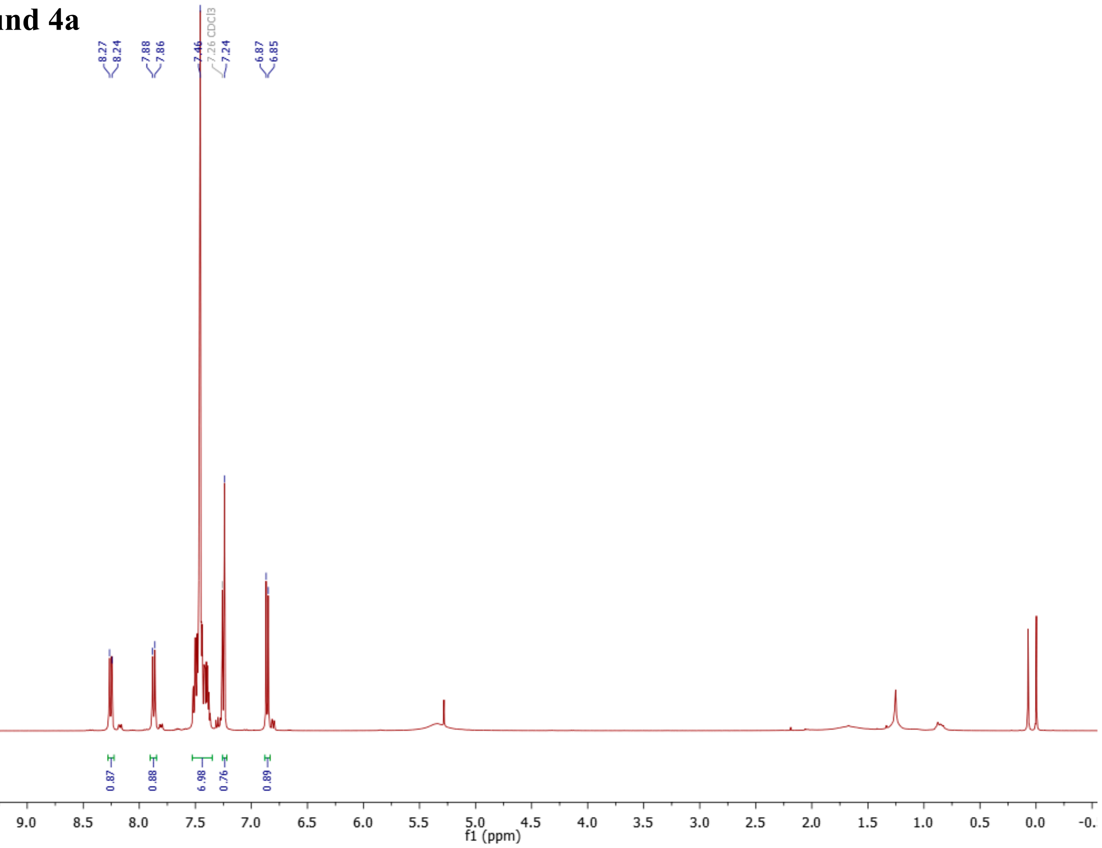

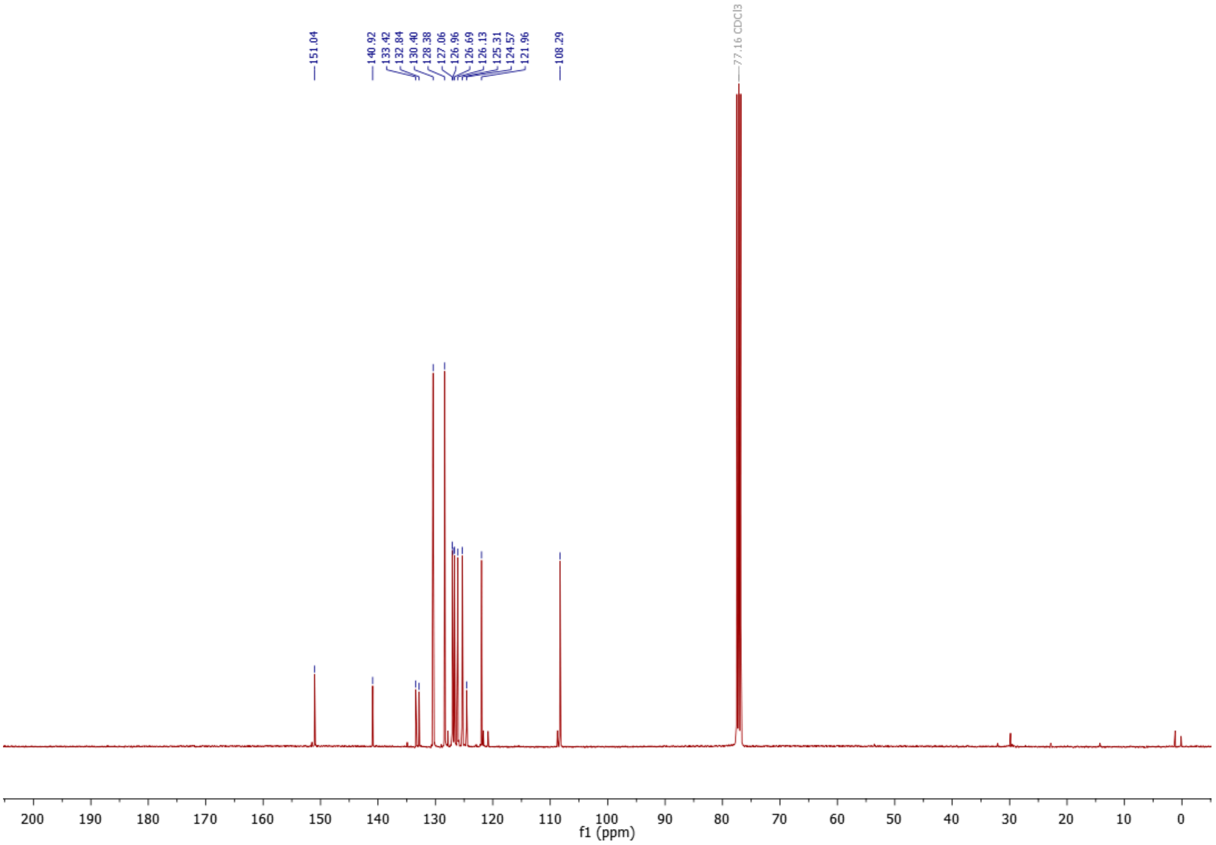

Figure S22. ^{1}H (400 MHz) and ^{13}C NMR (101 MHz) spectra of compound **4a** in $CDCl_3$.

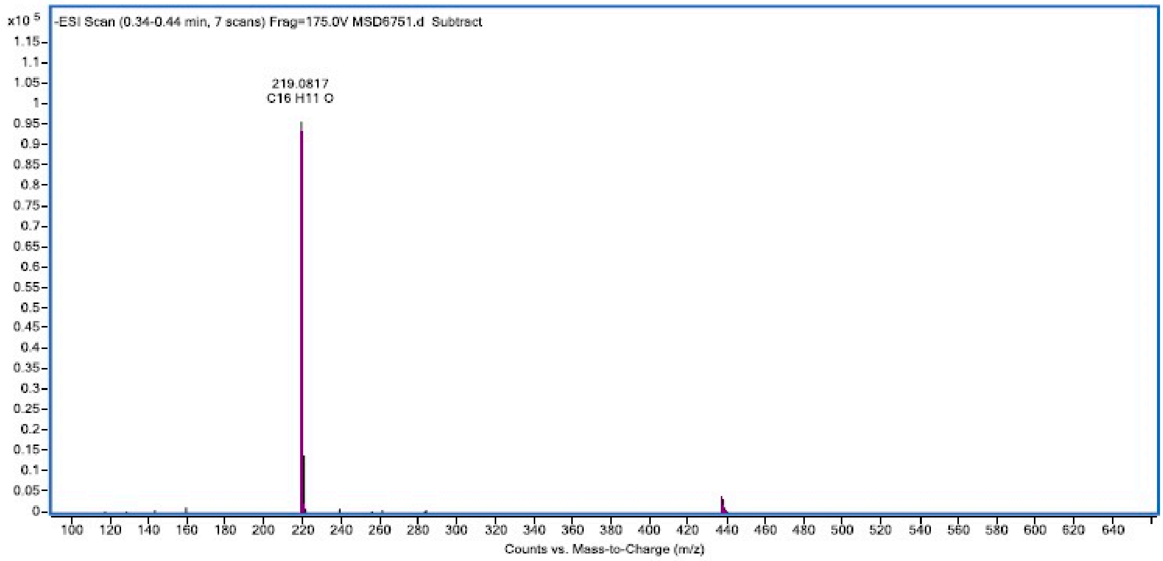

Figure S23. HR ESI-MS spectrum of compound **4a** in MeOH.

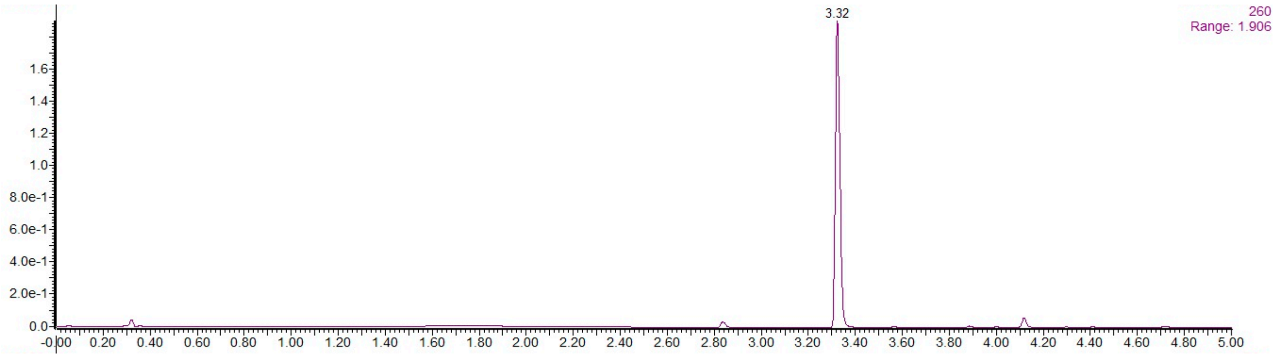

Figure S24. Reversed-phase HPLC chromatogram of compound **4a**.

Compound 5a

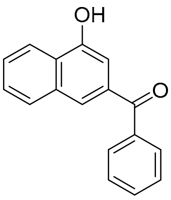

5a

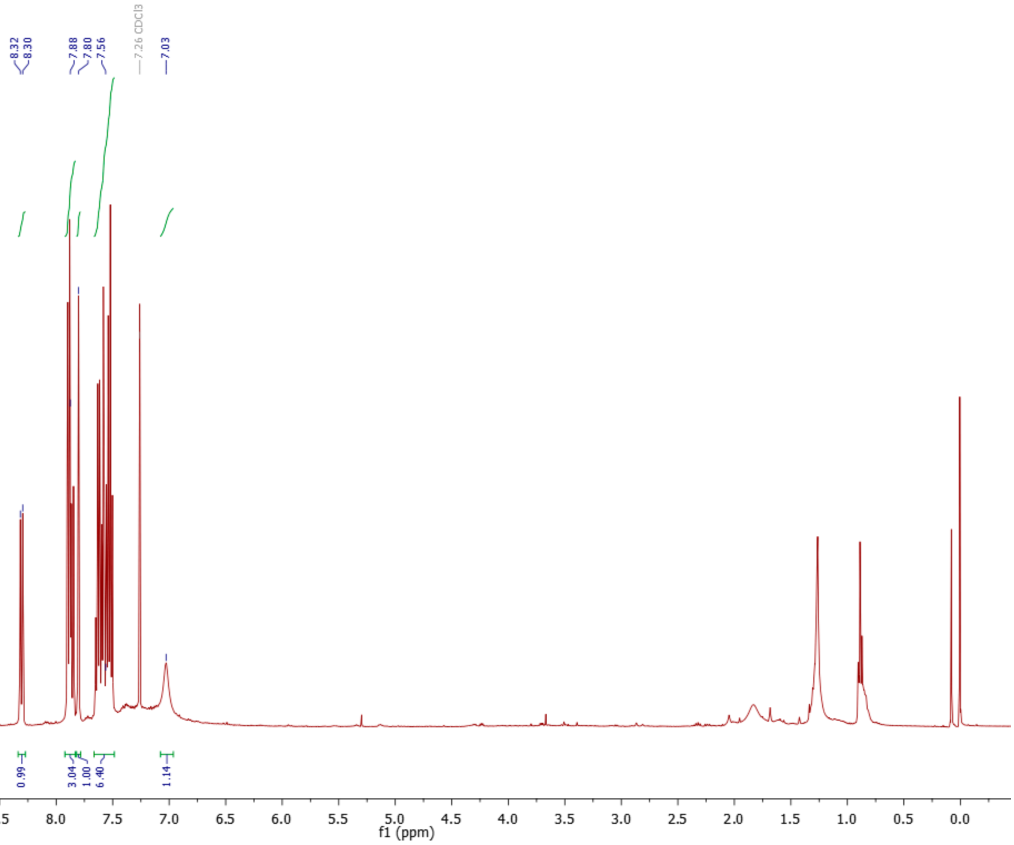

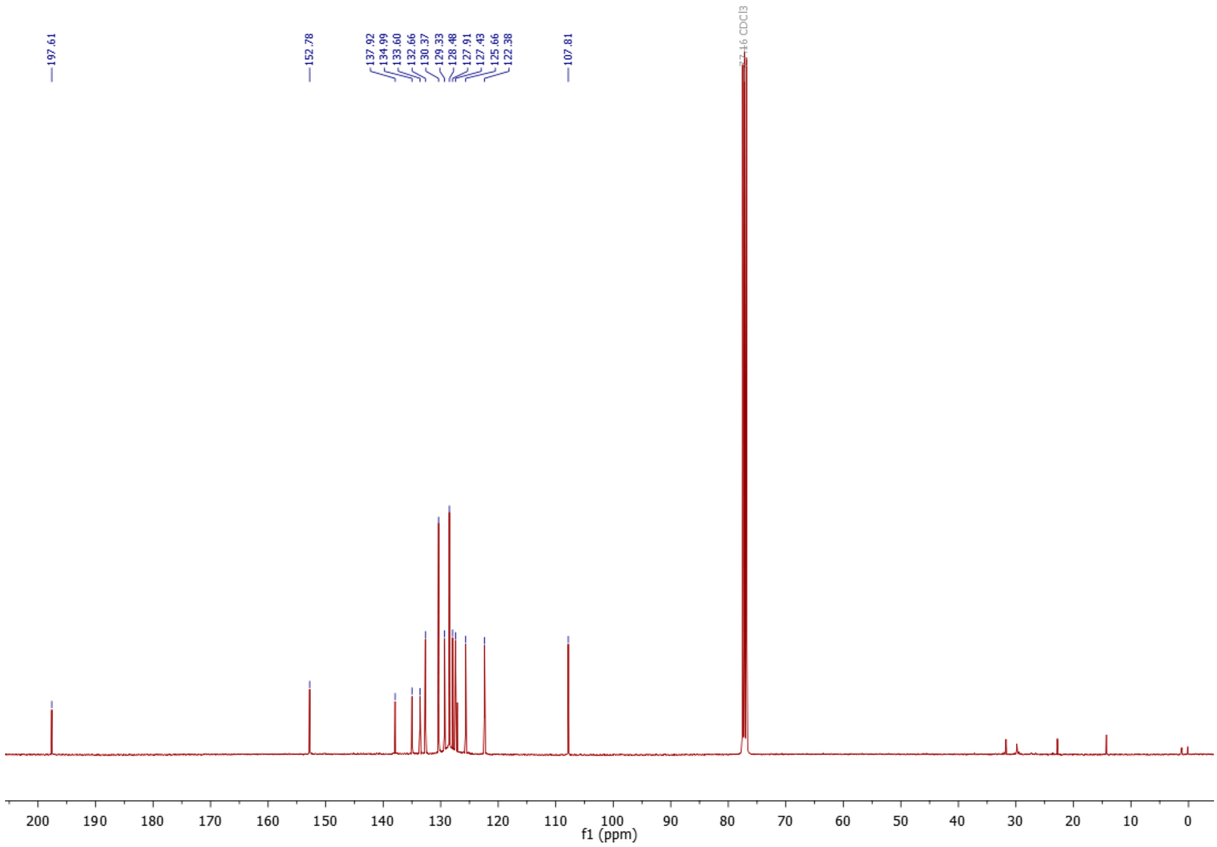

Figure S25. ^{1}H (400 MHz) and ^{13}C NMR (101 MHz) spectra of compound **5a** in $CDCl_3$.

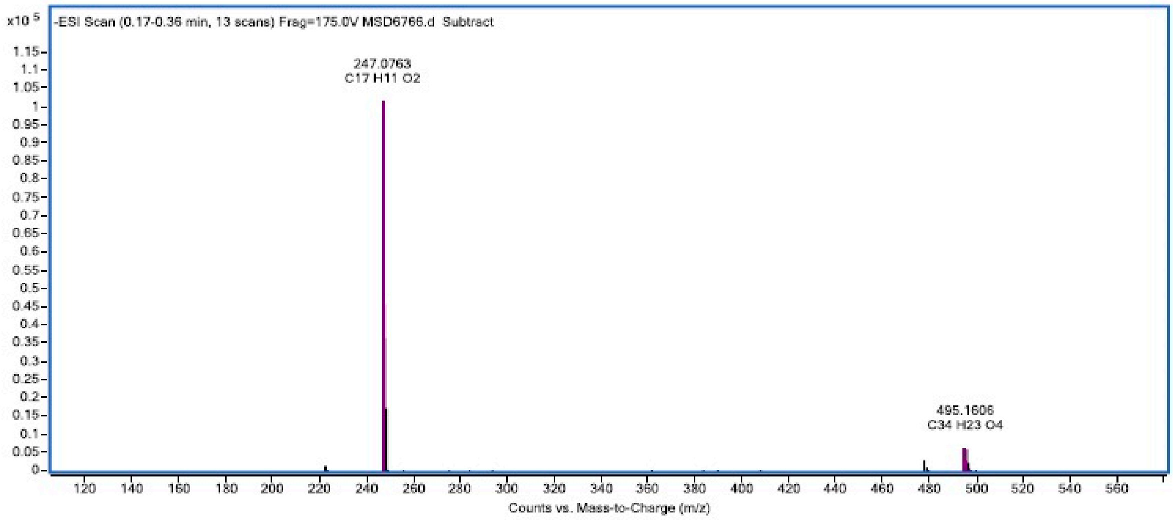

Figure S26. HR ESI-MS spectrum of compound **5a** in MeOH.

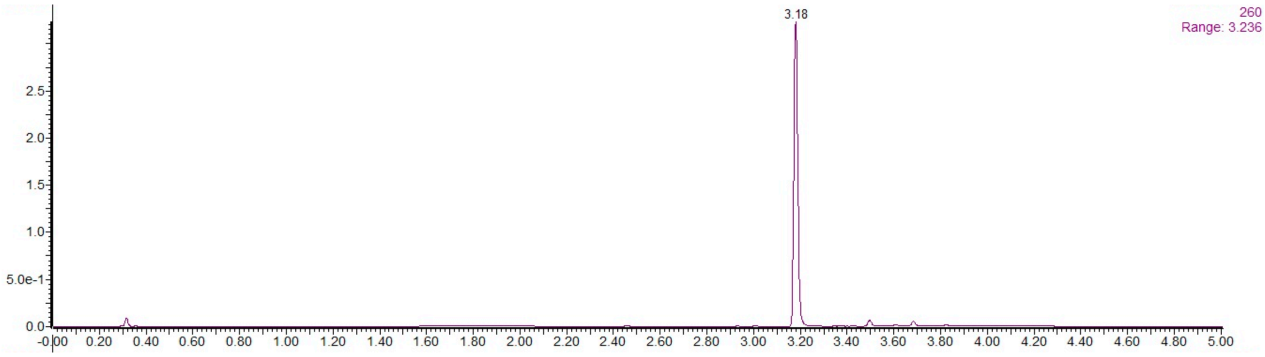

Figure S27. Reversed-phase HPLC chromatogram of compound **5a**.

^{1}H and ^{13}C NMR spectra, HR ESI-MS and HPLC analysis of compound 7.

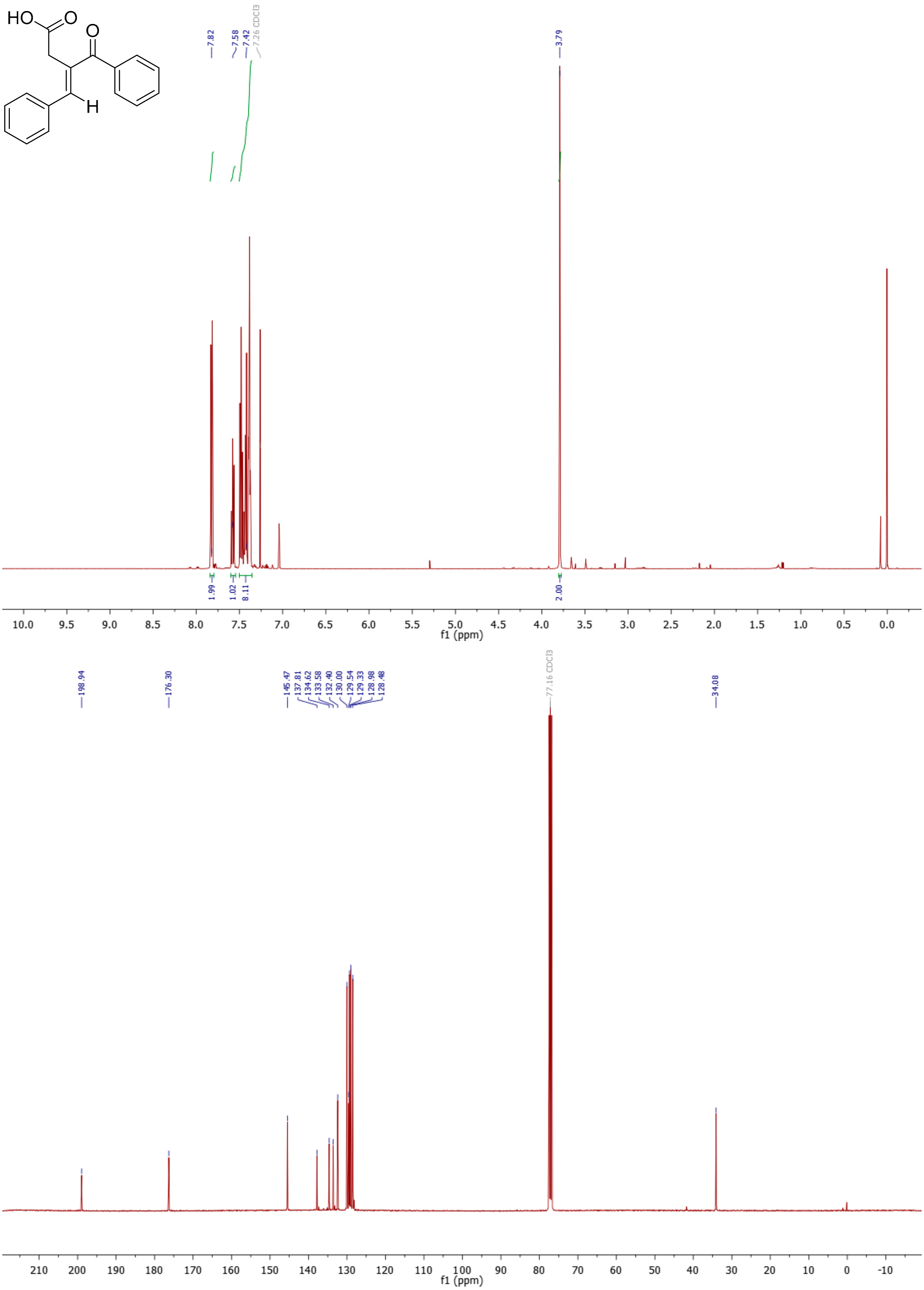

Figure S28. ^{1}H (400 MHz) and ^{13}C NMR (101 MHz) spectra of compound **7** in $CDCl_3$.

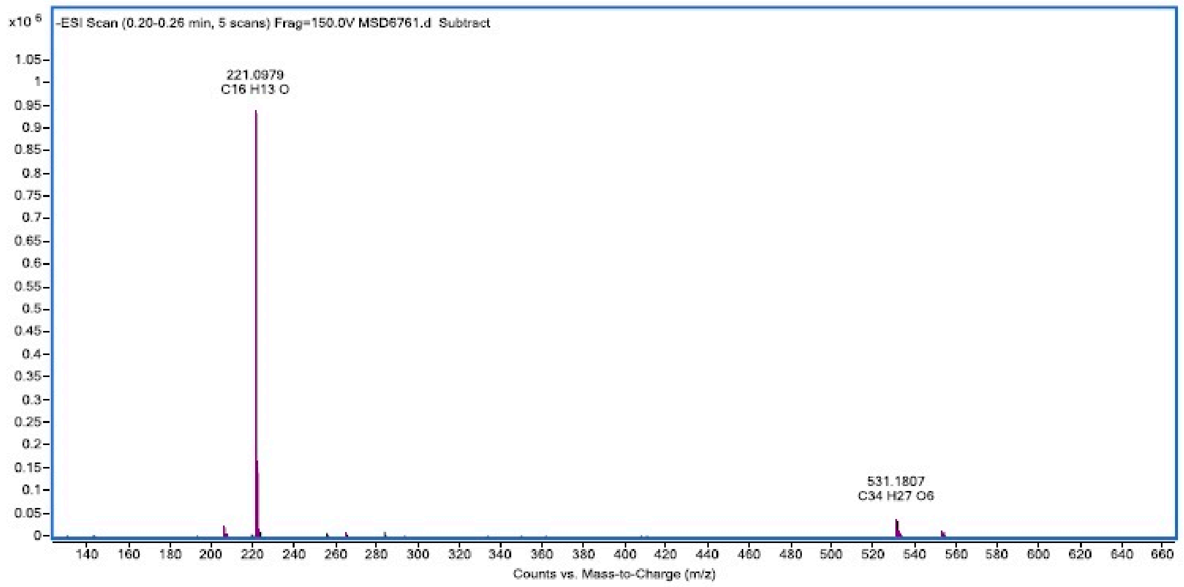

Figure S29. HR ESI-MS spectrum of compound **7** in MeOH.

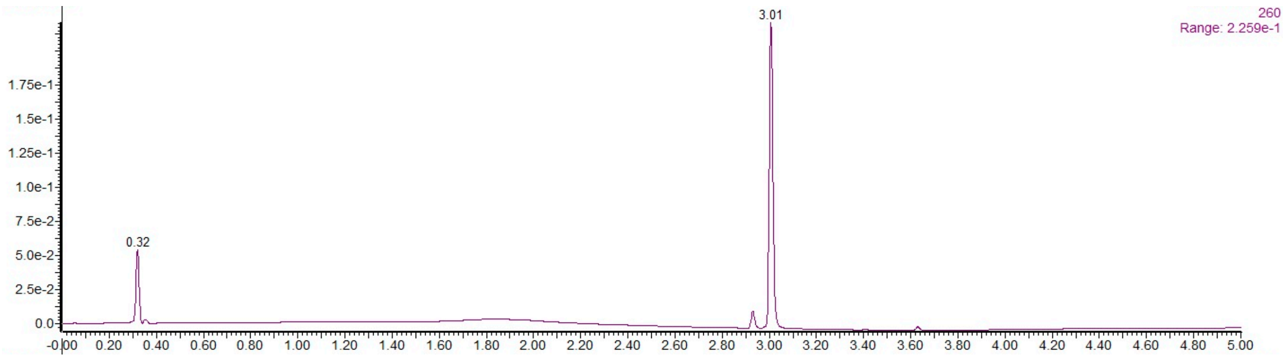

Figure S30. Reversed-phase HPLC chromatogram of compound **7**.
